# Supplementary material for: A Stem Cell Reporter for Investigating Pluripotency and Self-Renewal in the Rat
Source: Stem Cell Reports. 2020 Jan 2;14(1):154–66. doi: 10.1016/j.stemcr.2019.12.001 (PMC6962659; doi:10.1016/j.stemcr.2019.12.001)
Supplement: Document S1. Supplemental Experimental Procedures, Figures S1–S6, and Table S1–S6 [file mmc1.pdf]

Stem Cell Reports, Volume *14*

Supplemental Information

A Stem Cell Reporter for Investigating Pluripotency and Self-Renewal in the Rat

Stephen Meek, Jun Wei, Taeho Oh, Tom Watson, Jaime Olavarrieta, Linda Sutherland, Daniel F. Carlson, Angela Salzano, Tamir Chandra, Anagha Joshi, and Tom Burdon

Figure S1. (Related to Figure 1)

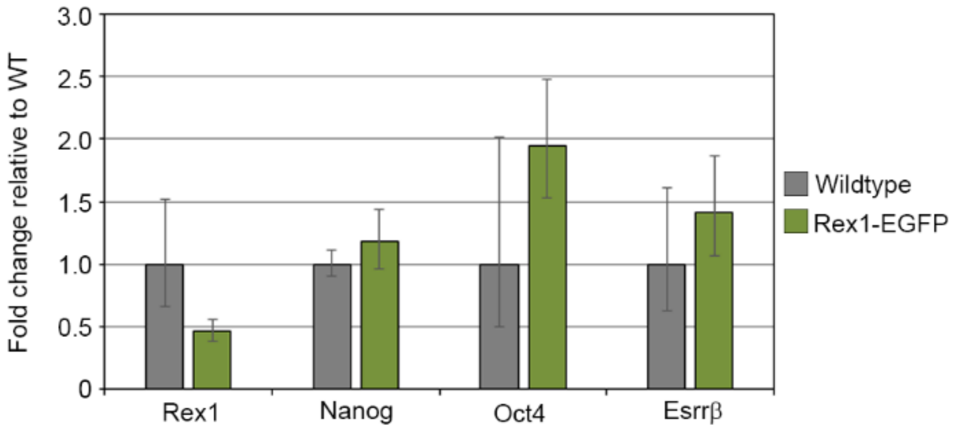

Figure S1. ESC gene expression in heterozygous *Rex1*-EGFP ESCs. qRT-PCR analysis for *Rex1* and the core ESC transcription factors Nanog, Oct4 and Esrrb in wild type (WT) and heterozygous *Rex1*-EGFP rESCs (mean and SD of three WT and four HET biological replicates).

Figure S2. (Related to Figure 2)

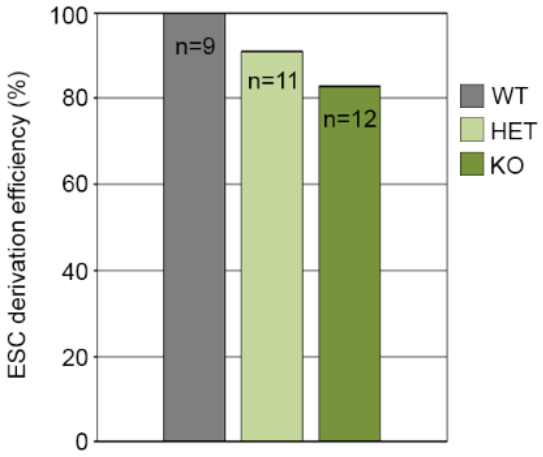

Figure S2. Derivation efficiency of *Rex1*-EGFP ESC lines.
Derivation efficiency of wild type (WT), heterozygous (HET) and knockout (KO) *Rex1*-EGFP ESCs from a total of nine, eleven and twelve E4.5 blastocysts respectively (all genotypes were derived from a total of four crosses).

Figure S3. (Related to Figure 4)

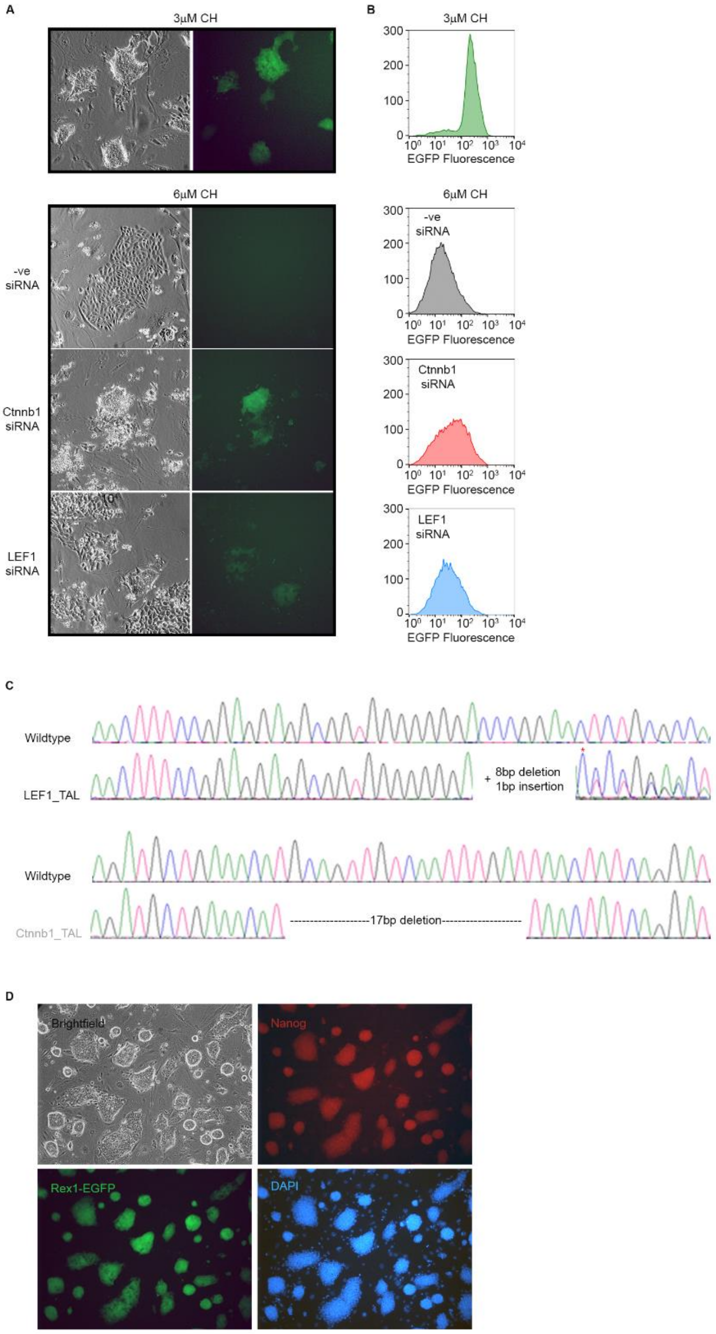

Figure S3. Sensitivity of *Rex1*-EGFP reporter during CHIR-induced differentiation and siRNA-mediated rescue. (A) Brightfield and fluorescent images of *Rex1*-EGFP rat ESCs cultured for four days in normal (3mM) and differentiating (6mM) rESC culture conditions following transfection with control (-ve), Ctnnb1 or LEF1 siRNAs. (magnification x100). (B) Flow cytometry analysis of *Rex1*-EGFP rESCs cultured for four days in normal (3mM) and differentiating (6mM) rESC culture conditions following transfection with control (-ve), Ctnnb1 or LEF1 siRNAs. (C) Sequence chromatograms of genomic DNA from wild type and TALEN-modified Ctnnb1 and LEF1 rESC clones showing frameshift-modified alleles (red asterisk highlights inserted base). (D) Brightfield, fluorescent (EGFP) and immunostained (Nanog) images of *Rex1*-EGFP rESC colonies cultured in 2iL. DAPI-stained cells are also shown. (magnification x100).

Figure S4. (Related Figure 5)

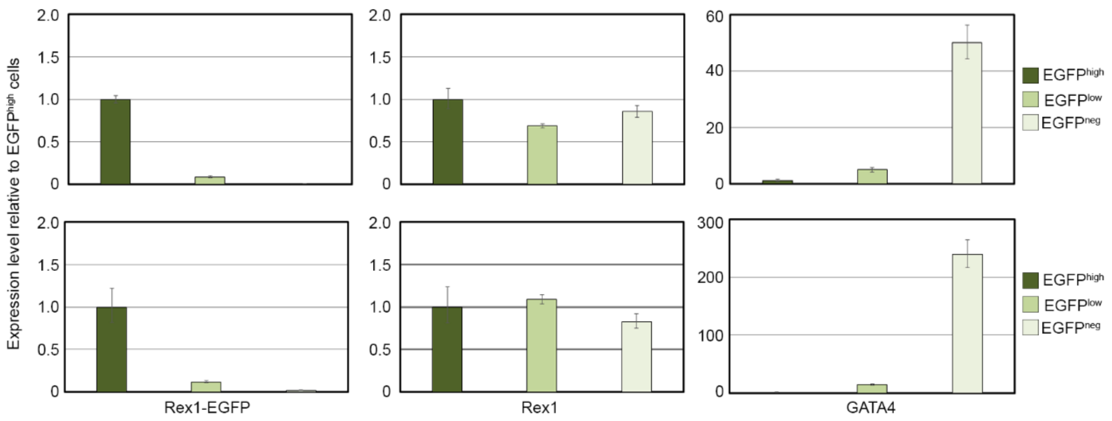

Figure S4. qRT-PCR analysis of FAC-sorted *Rex1*-EGFP rESCs.

qRT-PCR analysis for *Rex1*-EGFP, *Rex1* and *Gata4* in FAC-sorted *Rex1*-EGFP high, low and negative cells from two independent rESC lines (mean and SD of three technical replicates).

Figure S5. (Related to Figure 6)

A

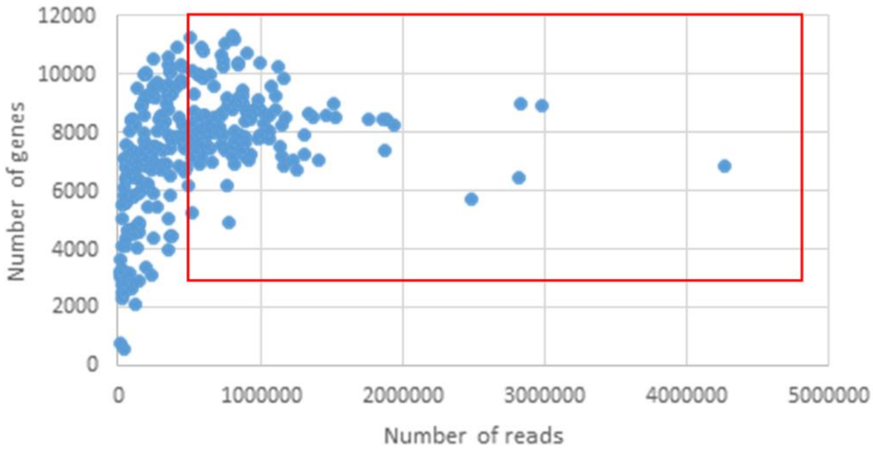

B

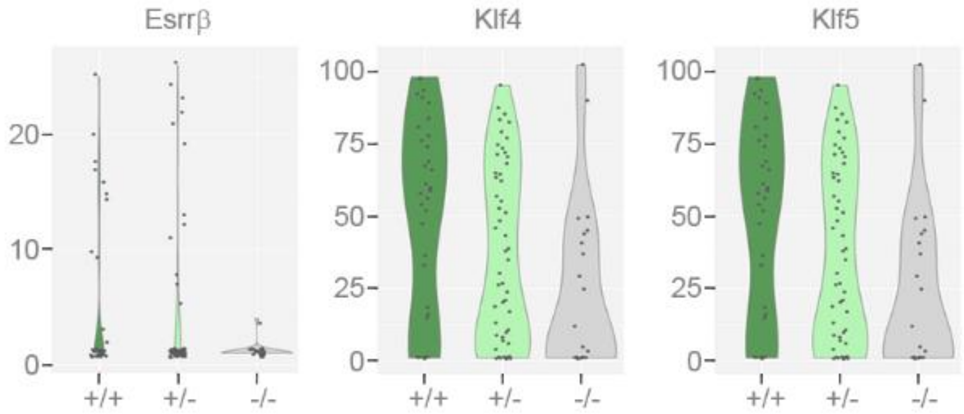

Figure S5. Single cell expression analysis of rESC. (A) Single cell RNA sequence quality control selection. Plot depicting the number of reads against the number of genes for each single cell sample. The cells in the red box were selected for downstream analysis. (B) Violin plots of *Esrrb*, *Klf4* and *Klf5* expression in *Rex1*$^{\text{mRNA+/EGFP+}}$, *Rex1*$^{\text{mRNA+/EGFP-}}$ and *Rex1*$^{\text{mRNA-/EGFP-}}$ cells.

Figure S6. (Related to Figure 6)

A

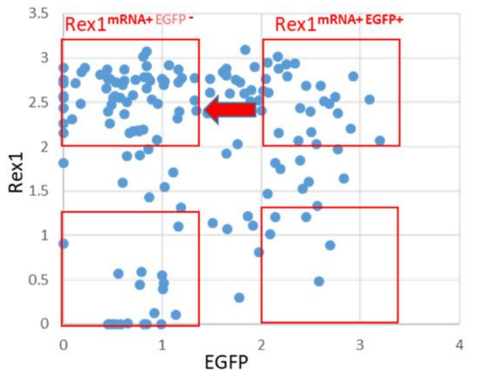

B

DOWN-REGULATED: Rps12, Fam120b, Zbtb45, Ube2s, Tmem160, Zfp428, Egln2, Hnrnpl, Spint2, Emc10, Ptov1, Nucb1, Prmt3, LOC100912195, Igf1r, Bnc1, Nup98, Taf10, Eif3f, Ubfd1, Plk1, Xpo6, Mapk3, Mvp, Utf1, Deaf1, Dhcr7, Dhcr7, Tmem134, Fam89b, Scyl1, Stx5, Tmem179b, Ints5, Incenp, Carnmt1, Uhrf2, Fam169a, LOC103689968, Hdgf, Hist2h2aa2, Hist2h2aa3, Ddx20, Gclm, Ssx2ip, Zzz3, Armc10, Bpgm, Slc25a26, Ptms, Rpl8, Qsox2, Ttf1, LOC100912604, Celf1, Atg13, Dnajc17, Ndufaf5, Map1lc3a, Scand1, Dnttip1, Dnttip1, Ube2c, Prpf6, Nudt2, Arhgef39, Clta, Igfbpl1, LOC108351137, Tmem39b, Cep85, Pink1, Ctnnbip1, Prkcz, Maoa, Dkc1, Fez2, Gckr, Ppm1g, Rps10l1, Baz1a, Rpl6-ps1, Glrx5, Crip1, Myl6, Fzr1, Gadd45b, Sppl2b, Sf3a2, Med16, Zfp472, Ccdc59, Gns, Shmt2, Trmt12, Grina, Gpaa1, Fbxl6, Pim3, Zcrb1, LOC688906, Tuba1c, Atg101, Atg4d, Ilf3, Rp9, Sidt2, Alas1, Smarcc1, Rpsa, Rpl36, Safb, Mpnd, Tjap1, Tmem63b, Ptpn18, Rnf149, Tex30, Sgo2, RGD1562399, Mreg, Cnppd1, Abcb6, Wdfy1, Ilkap, Crlf2, Tmem165, LOC257642, LOC257642, Ctbp1, Rasl10a, Mrps24, Nudcd3, Cobl, Wdr92, Insig2, Fmod, Uhmk1, Psen2, Tmem63a, Pdpk1, Eme2, Gnptg, Chtf18, Fam173a, Jmjd8, Stub1, Hba-a3, Hba-a2, Mpg, Ergic1, RGD1564698, Hnrnpab, Cdkn2aipnl, Rnf187, LOC108348082, Sco1, Aurkb, C1qbp, Ube2g1, Spag5, Trim25, Fam117a, Top2a, Nt5c3b, Rab5c, Lsm12, Cdc42ep4, Recql5, Galk1, Slc38a10, Faap100, Mafg, LOC108348081, Ccnb1ip1, Zfp219, Lrp10, Psmb5, Cdca2, Wdfy2, R3hcc1, Polr3d, B4galt7, Rab24, Tspan17, AC120310.1, LOC679342, Mcur1, Lyrm4, Hist1h1d, Hist1h3b, Hist1h1b, Wac, Rbm17, Ccser2, Ocel1, Fkbp8, Sugp2, Armc6, Gapdh-ps2, Dusp26, Coprs, Upf3a, Lamp1, Osbpl11, Cep19, Slc25a1, Rock1, Sil1, Ccdc112, Heatr3, Ccdc130, RGD1564093, Prmt9, Ctcf, Pskh1, Prmt7, Cog8, Aars, Gtf2h4, RT1-CE16, Ddah2, Bag6, Kifc1, RGD1564450, Snrpc, Trappc5, Gtf3a, Rac1, Cops6, Tmem248, Ccdc92, Plbd2, Gcn1l1, Unc119b, Iscu, Pitpnb, Chchd10, Tbx3, eGFP. **UP-REGULATED**: Ndufa4, Fubp3, LOC102557117, Prdx1, Srsf7, Map3k12, Sumo1, H3f3b, Calm2, Jund, Naa50, LOC102550729, Bud31, Mt-nd2

C

GO: Biological Process

	ID	Name	Source	pValue	Bonferroni	Genes from Input	Genes in Annotation
1	GO:0051276	chromosome organization		1.354E-8	4.491E-5	39	1189
2	GO:0007059	chromosome segregation		7.488E-7	2.483E-3	17	333
3	GO:0016071	mRNA metabolic process		6.975E-6	2.313E-2	23	664

D

Chromosome organisation GO:0051276

	Entrez Gene ID	Gene Symbol	Gene Name
1	3619	INCENP	inner centromere protein
2	55352	COPRS	coordinator of PRMT5 and differentiation stimulator
3	7270	TTF1	transcription termination factor 1
4	7786	MAP3K12	mitogen-activated protein kinase kinase kinase 12
5	51322	WAC	WW domain containing adaptor with coiled-coil
6	646	BNC1	basonuclin 1
7	8337	HIST2H2AA3	histone cluster 2 H2A family member a3
8	6294	SAFB	scaffold attachment factor B
9	8358	HIST1H3B	histone cluster 1 H3 family member b
10	9400	RECQL5	RecQ like helicase 5
11	1736	DKC1	dyskerin pseudouridine synthase 1
12	151246	SGO2	shugoshin 2
13	54496	PRMT7	protein arginine methyltransferase 7
14	6881	TAF10	TATA-box binding protein associated factor 10
15	5347	PLK1	polo like kinase 1
16	115426	UHRF2	ubiquitin like with PHD and ring finger domains 2
17	10980	COPS6	COP9 signalosome subunit 6
18	7917	BAG6	BCL2 associated athanogene 6
19	3833	KIFC1	kinesin family member C1
20	11065	UBE2C	ubiquitin conjugating enzyme E2 C
21	4928	NUP98	nucleoporin 98
22	723790	HIST2H2AA4	histone cluster 2 H2A family member a4
23	80218	NAA50	N(alpha)-acetyltransferase 50, NatE catalytic subunit
24	10615	SPAG5	sperm associated antigen 5
25	415116	PIM3	Pim-3 proto-oncogene, serine/threonine kinase
26	2968	GTF2H4	general transcription factor IIH subunit 4
27	10664	CTCF	CCCTC-binding factor
28	11177	BAZ1A	bromodomain adjacent to zinc finger domain 1A
29	3007	HIST1H1D	histone cluster 1 H1 family member d
30	3009	HIST1H1B	histone cluster 1 H1 family member b
31	6599	SMARCC1	SWI/SNF related, matrix associated, actin dependent regulator of chromatin subfamily c member 1
32	3021	H3F3B	H3 histone family member 3B
33	1487	CTBP1	C-terminal binding protein 1
34	10196	PRMT3	protein arginine methyltransferase 3
35	5595	MAPK3	mitogen-activated protein kinase 3
36	57820	CCNB1IP1	cyclin B1 interacting protein 1
37	7153	TOP2A	DNA topoisomerase II alpha
38	65018	PINK1	PTEN induced kinase 1
39	9212	AURKB	aurora kinase B

E

Chromosome segregation GO:0007059

	Entrez Gene ID	Gene Symbol	Gene Name
1	3619	INCENP	inner centromere protein
2	157313	CDCA2	cell division cycle associated 2
3	9400	RECQL5	RecQ like helicase 5
4	151246	SGO2	shugoshin 2
5	5347	PLK1	polo like kinase 1
6	7917	BAG6	BCL2 associated athanogene 6
7	3833	KIFC1	kinesin family member C1
8	64793	CEP85	centrosomal protein 85
9	11065	UBE2C	ubiquitin conjugating enzyme E2 C
10	4928	NUP98	nucleoporin 98
11	80218	NAA50	N(alpha)-acetyltransferase 50, NatE catalytic subunit
12	10615	SPAG5	sperm associated antigen 5
13	10664	CTCF	CCCTC-binding factor
14	63922	CHTF18	chromosome transmission fidelity factor 18
15	57820	CCNB1IP1	cyclin B1 interacting protein 1
16	7153	TOP2A	DNA topoisomerase II alpha
17	9212	AURKB	aurora kinase B

Figure S6. Genes differentially expressed between *Rex1*$^{\mathbf{mRNA+EGFP+}}$ and *Rex1*$^{\mathbf{mRNA+EGFP-}}$ cell populations. (**A**) Scatter plot identifying the *Rex1*$^{\mathbf{mRNA+EGFP+}}$ and *Rex1*$^{\mathbf{mRNA+}\mathrm{EGFP-}}$ cell populations**.** (**B**) List of genes differentially expressed between the *Rex1*$^{\mathbf{mRNA+EGFP+}}$ and *Rex1*$^{\mathbf{mRNA+}\mathrm{EGFP-}}$ cells. (**C**) Three biological processes most highly enriched in the differentially expressed genes. (**D**) List of differentially expressed genes associated with chromosome organisation. (**E**) List of differentially expressed genes associated with chromosome segregation.

Table S1. Chimaera formation and germline transmission efficiency. (Related to Figure 1)

Cell line	Genotype	Pups born	Chimaeras	GLT transmission
DAK31	Wild type	62	10M/11F (34%)	4/10 (40%)
E3	targeted	32	7M/6F (41%)	2/7 (29%)

Table S2. Viability of *Rex1* null rats. (Related to Figure 1)

Pups born	Wild type	Heterozyous	Homozygous
205[#]	41 (20%)	122 (60%)	42 (20%)

[#] pups born from six breeding pairs.

Table S3. *Rex1* null male rat fertility. (Related to Figure 1)

Male genotype	Stage	No. of litters	No. of embryos	Av. Litter size
Wild type (n=3)	Mid-gestation	5	40	8.0
Wild type (n=2)	E4.5	3	10	3.3
	Total	8	50	6.2
Rex1-null (n=4)	Mid-gestation	5	28	5.6
Rex1-null (n=4)	E4.5	4	27	6.7
	Total	9	55	6.1

Table S4. qRT-PCR primer list. (Related to Experimental Procedures)

**EGFP Forward**	**ACGTAAACGGCCACAAGTTC**
**EGFP Reverse**	**AAGTCGTGCTGCTTCATGTG**
**Esrrb Forward**	**AGGCTTGCAAGGCGTTCTT**
**Esrrb Reverse**	**AGGACTTGCGCCTCCGTTT**
**Gapdh Forward**	**ATGACTCTACCCACGGCAAG**
**Gapdh Reverse**	**TGGGTTTCCCGTTGATGACC**
**Gata4 Forward**	**GCGGCCTCTACATGAAGCTC**
**Gata4 Forward**	**TTCCGTTTTCTGGTTTGAATC**
**Nanog Forward**	**TACCTCAGCCTCCAGCAGAT**
**Nanog Reverse**	**GCAATGGATGCTGGGATACT**
**Oct4 Forward**	**GAAGTTGGAGAAGGTGGAACC**
**Oct4 Reverse**	**GTGTACCCCAAGGTGATCCTC**
**Rex1 Forward**	**GATTTCAACTTGCGCACCC**
**Rex1 Reverse**	**CTCTTCTCGCAGCCATCAAAA**
**Sox2 Forward**	**AACCCCAAGATGCACAACTC**
**Sox2 Reverse**	**CTTGGCCTCGTCGATGAAC**
**Tsix Forward**	**GTATCCACAGCCCCGATG**
**Tsix Reverse**	**ACCTCGGATACCTGCGTTT**

Table S5. Number of *Rex1*-EGFP FAC-sorted single cells selected for each population after quality control. (Related to Figure 6)

Plate	$EGFP^{neg}$	$EGFP^{low}$	$EGFP^{high}$
1	16(32)	28(32)	8(32)
2	23(32)	27(32)	10(32)
3	10(32)	16(32)	13(32)
4#	43(96)		

() Total number of cells collected.

Replated *Rex1*-$EGFP^{low}$ cells.

Table S6. Number of cells selected based on EGFP fluorescence and mRNA expression (*Rex1* and EGFP). (Related to Figure 6)

	$EGFP^{neg}$	$EGFP^{low}$	$EGFP^{high}$	$EGFP^{low\text{-}replated}$
Rex1$^{mRNA+/EGFP+}$ (35)	2	19	14	0
Rex1$^{mRNA+/EGFP-}$ (78)	15	37	5	22
Rex1$^{mRNA-/EGFP-}$ (24)	11	0	1	12

Supplemental Experimental Procedures

Chimaera Generation

Rat blastocysts at E4.5 days post-coitum were collected by noon on the day of injection and cultured for 2–3 hours in KSOM embryo culture medium to ensure cavitation. Cells were disaggregated in TVP, pelleted in N2B27 and pre-plated on gelatin-coated tissue culture plastic in 2iL for 45-60 minutes. Non-attached cells were pelleted and resuspended in N2B27 containing 20mM HEPES buffer then kept on ice prior to injection. Blastocysts were injected with 10–12 cells, then transferred into the uteri of pseudopregnant Sprague Dawley rats.

Genotyping *Rex1*-EGFP rats

150ng of genomic DNA was amplified using a single PCR reaction containing three oligonucleotides designed to identify the wild type (REX5'interFOR2–GTGGATGTCAGGACAATCTGAG, REX5'wtREV2-CCACTTGTCTTTGCCATTTTCT) and targeted (REX5'interFOR2-GTGGATGTCAGGACAATCTGAG, REXgfpREV–GGACTTGAAGAAGTCGTGCTG) *Rex1* alleles. The PCR was performed using NEB Q5 HotStart Taq Polymerase under the following conditions; 98°C for 1 minute, followed by 32 cycles of 98°C for 10s, 65°C for 30s and 72°C for 1 minute with a final extension of 72°C for 10 minutes. Products were visualised with ethidium bromide on a 2% TAE agarose gel. Expected sizes for wild-type and targeted alleles are 292bp and 524bp respectively.

Sexing rats and rESCs

150ng of genomic DNA was amplified using a single PCR reaction containing four oligonucleotides. The presence of a Y chromosome was determined using oligonucleotides designed to the rat *Sry* gene (For-AAGCCTTACAGAAGCCGAAA, Rev-TACAGTTTTGTTGAGGCAACT) and oligonucleotides designed to the rat *Sox2* gene (For-ATGATGGAGACGGAGCTGAA, Rev-CTCCGGGAAGCGTGTACTTA) were used as an internal control. The PCR was performed using NEB Q5 HotStart Taq Polymerase under the following conditions; 98°C for 1 minute, followed by 32 cycles of 98°C for 10s, 65°C for 30s and 72°C for 30s with a final extension of 72°C for 10 minutes. Products were visualised with ethidium bromide on a 2% TAE agarose gel. Expected sizes for *Sry* and *Sox2* PCR products are 165bp and 385bp respectively.

Southern blotting

Eight to ten micrograms of genomic DNA were digested with 200 units of restriction enzyme at 37°C for 30 hours. The resulting DNA fragments were resolved on a 0.7% TAE agarose gel overnight at 25V. The DNA fragments were UV-nicked prior to transfer to Hybond N + Nylon membrane (GE Healthcare, RPN203B) as described in the manufacturer's instructions. Following transfer, the DNA was UV cross-linked on to the membrane. Probes were prepared by PCR amplification of *Rex1* sequence flanking the homology arms to generate a 595pb 5' probe and a 360bp 3' probe (5' probe sequence TAGAATTCATAAGACGTTGG to TTTGGCATGCACTTATTTA and 3' probe sequence TTCCACCCCCTCCCTCATGC to TGGTTACATTGTGTTTCTGC). 25ng of probe DNA was radioactively labelled with α–dCTP P^{32} using High Prime (Roche, 11 585 592 001), then hybridised to the membrane overnight at 65°C in Church solution containing 10μg/ml sonicated Herring Sperm DNA and 10μg/ml tRNA. Non-specifically bound probe was removed by washing in 2xSSC/0.1% (w/v) SDS at 65°C. The membrane was exposed to Kodak Biomax MS film at -80°C.

TALEN construction

The *β-catenin* TALEN pair (left target – GCTGAAACATGCAGTT, right target - ATCAGGATGACGCGG) were designed to exon 3 and kindly generated and supplied by Dan Carlson (Recombinetics, Inc.). The *Lef1* TALEN pair (left target – CCGGAGGAGGCGGTG, right target - TGCGCCACCGATGAG) were designed to exon 1 and generated by Golden Gate cloning. All TALENs were designed using the TALE-NT software (https://tale-nt.cac.cornell.edu/node/add/talen) and assembled using methods described in Sakuma et al. The TALENs were cloned into pCAG-T7 TALEN (Sangamo)-FokI-ELD-Destination and pCAG-T7-TALEN (Sangamo)-FokI-KKR-Destination expression plasmids. Rat ESCs were transfected at a density of 0.5x10^{4}/cm^{2} with 240ng left and right TALEN expression plasmid using Lipofectamine LTX (2.25μl LTX + 0.75μl PLUS reagent, Invitrogen) according to manufacturer's instructions. Transfection reagents were removed 16 hours post-transfection and the cells cultured for a further 3 days prior to imaging and flow cytometry analysis.

siRNA knockdown

Rat ESCs were transfected at a density of 0.5x10^{4}/cm^{2} with *Ctnnb1* (Ambion, s136459), *Lef1* (s139217) or negative control (Ambion, 4390846) siRNA at a final concentration of 50nM using Lipofectamine LTX (2.25μl

LTX + 0.75µl PLUS reagent, Invitrogen) according to manufacturer's instructions. Transfection reagents were removed 16 hours post-transfection and the cells cultured for a further 3 days prior to imaging and flow cytometry analysis.

Bioinformatic Analysis

The sequence files were first checked through FASTQC and then the sequencing reads were mapped to rat genome assembly Rnor_6.0 using Kallisto aligner (Bray et al., 2016). The gene counts were generated using HT-seq. We used a stringent criterion to select individual cells based on the sequencing depth (over 500K reads) and the total number of genes (over 3000 genes) (Figure S6). This resulted into a total of 49 $EGFP^{neg}$, 71 $EGFP^{low}$ and 31 $EGFP^{high}$ cells selected for further downstream analysis (Table S5). We also validated that all selected cells were obtained using ERCC count and MT read filter as well. The data for selected samples was normalised using Scran (Lun et al., 2016). The batch correction was performed using ComBat. The differentially expressed gene sets were obtained using scde (Kharchenko et al., 2014). We noted the inconsistency between EGFP and *Rex1* mRNA expression across cells (Figure 6). We therefore grouped cells based on EGFP and *Rex1* mRNA expression into three groups $\textit{Rex1}^{mRNA+/EGFP+}$ (log expression value > 2), $\textit{Rex1}^{mRNA+/EGFP-}$ and $\textit{Rex1}^{mRNA-/EGFP-}$(log expression value < 1.3). Table S6 shows the overlap of cells selected based on EGFP activity and mRNA expression (*Rex1* and EGFP) groupings. All analysis was performed in R including generation of PCA plots and box plots.

Gene list enrichment analysis was performed using the ToppFun application within the ToppGene Suite (https://toppgene.cchmc.org/).

Ethics statement

Animal work conformed to guidelines for animal husbandry according to the UK Home Office and approval by the Roslin Institute Animal Ethics Committee. Animals were naturally mated and sacrificed under schedule 1, procedures that do not require specific Home Office approval.

Supplemental References

Bray, N.L., Pimentel, H., Melsted, P., and Pachter, L. (2016). Near-optimal probabilistic RNA-seq quantification. Nat. Biotechnol. *34*, 525–527.

Kharchenko, P. V, Silberstein, L., and Scadden, D.T. (2014). Bayesian approach to single-cell differential expression analysis. Nat. Methods *11*, 740–742.

Lun, A.T.L., Bach, K., and Marioni, J.C. (2016). Pooling across cells to normalize single-cell RNA sequencing data with many zero counts. Genome Biol. *17*, 75.
