# Supplementary material for: A Stem Cell Reporter for Investigating Pluripotency and Self-Renewal in the Rat
Source: Stem Cell Reports. 2020 Jan 2;14(1):154–66. doi: 10.1016/j.stemcr.2019.12.001 (PMC6962659; doi:10.1016/j.stemcr.2019.12.001)
Supplement: Document S2. Article plus Supplemental Information [file mmc2.pdf]

Stem Cell Reports
Resource

ISSCR
OPEN ACCESS

A Stem Cell Reporter for Investigating Pluripotency and Self-Renewal in the Rat

Stephen Meek,[1,5] Jun Wei,[1,2,5] Taeho Oh,[1] Tom Watson,[1] Jaime Olavarrieta,[1] Linda Sutherland,[1] Daniel F. Carlson,[3] Angela Salzano,[4] Tamir Chandra,[4] Anagha Joshi,[1] and Tom Burdon[1,*]
[1]The Roslin Institute and R(D)VS, University of Edinburgh, Easter Bush, Midlothian, EH25 9RG, UK
[2]iRegene Therapeutics, C6-522, 666 Gaoxin Avenue, Wuhan, 430070, China
[3]Recombinetics Inc., 1246 University Avenue W, St. Paul, MN 55125, USA
[4]MRC Unit for Human Genetics, Institute of Genetics and Molecular Medicine, The University of Edinburgh, Western General Hospital, Crewe Road South, Edinburgh, EH4 2XU, UK
[5]Co-first author
*Correspondence: tom.burdon@roslin.ed.ac.uk
https://doi.org/10.1016/j.stemcr.2019.12.001

SUMMARY

Rat embryonic stem cells (rESCs) are capable of contributing to all differentiated tissues, including the germ line in chimeric animals, and represent a unique, authentic alternative to mouse embryonic stem cells for studying stem cell pluripotency and self-renewal. Here, we describe an EGFP reporter transgene that tracks expression of the benchmark naive pluripotency marker gene *Rex1* (*Zfp42*) in the rat. Insertion of the EGFP reporter gene downstream of the *Rex1* promoter disrupted *Rex1* expression, but REX1-deficient rESCs and rats were viable and apparently normal, validating this targeted knockin transgene as a neutral reporter. The *Rex1*-EGFP gene responded to self-renewal/differentiation factors and validated the critical role of β-catenin/LEF1 signaling. The stem cell reporter also allowed the identification of functionally distinct sub-populations of cells within rESC cultures, thus demonstrating its utility in discriminating between cell states in rat stem cell cultures, as well as providing a tool for tracking *Rex1* expression in the rat.

INTRODUCTION

Embryonic stem cells (ESCs) are pluripotent, immortal, embryo-derived cell lines that can differentiate into all cells of a developing embryo, including the germ cells, and can be used to introduce genetic modifications through the germline (Doetschman et al., 1987; Evans and Kaufman, 1981; Hooper et al., 1987; Martin, 1981; Thomas and Capecchi, 1987). Although mouse ESCs have been available for more than 30 years, the application of this technology to other experimental animals has proved challenging. Nonetheless, recent advances in understanding the control of self-renewal in ESCs and the development of rationally designed culture systems enabled the first derivation of authentic rat ESCs (Buehr et al., 2008; Li et al., 2008; Ying et al., 2008). Historically, the laboratory rat has been a preferred research animal in many areas of biomedical investigation, including the cardiovascular system and the brain, and this breakthrough in stem cell technology provided a new approach to generate targeted genetic models in this important and useful experimental animal (Meek et al., 2017; Tong et al., 2010). Rat ESCs (rESCs) also provide a unique alternative to mouse ESCs with which to investigate mechanisms regulating pluripotency and self-renewal (Chen et al., 2013; Meek et al., 2013).

Standard mouse ESC culture conditions containing serum and leukemia inhibitory factor (LIF) do not support rESC culture (Buehr et al., 2003, 2008). However, a two-inhibitor (2i) culture system developed for mouse ESCs, which uses small molecule inhibitors to suppress mitogen-activated protein kinase kinase (MEK1/2) and glycogen synthase kinase 3 (GSK3) activity in conjunction with the cytokine LIF, allows the continuous proliferation of rESCs in culture (Buehr et al., 2008; Li et al., 2008; Ying et al., 2008). Rat ESCs are derived efficiently *de novo* from blastocysts using this 2i+LIF culture medium, but rESC lines are typically less stable than their mouse counterparts under conditions of clonal expansion and continuous culture (Blair et al., 2012; Meek et al., 2010). This instability can be mitigated to some extent by titrating the level of GSK3 inhibition, to limit the prodifferentiative actions of β-catenin in association with the transcription factor LEF1, which is highly expressed in rESCs (Chen et al., 2013; Meek et al., 2013). Understanding the molecular basis of the different responses of these two demonstrably pluripotent ESCs (that efficiently colonize embryos to generate chimeric animals) affords valuable insights into how signaling and intrinsic mechanisms combine to control pluripotency and differentiation in early embryonic development.

Fluorescent stem cell reporter genes provide accurate and sensitive feedback on the state of the cells in live cultures, and are useful and important tools for studying the behavior of stem cells and their derivatives. A valuable ESC reporter gene in this regard is the ESC-associated transcription factor REX1/ZFP42, which is highly expressed in the naive ESCs, the cell type captured *in vitro* in 2i+LIF cultures that most closely represents pluripotent stem cells in the preimplantation blastocyst embryo (Boroviak et al.,

This is an open access article under the CC BY license (http://creativecommons.org/licenses/by/4.0/).

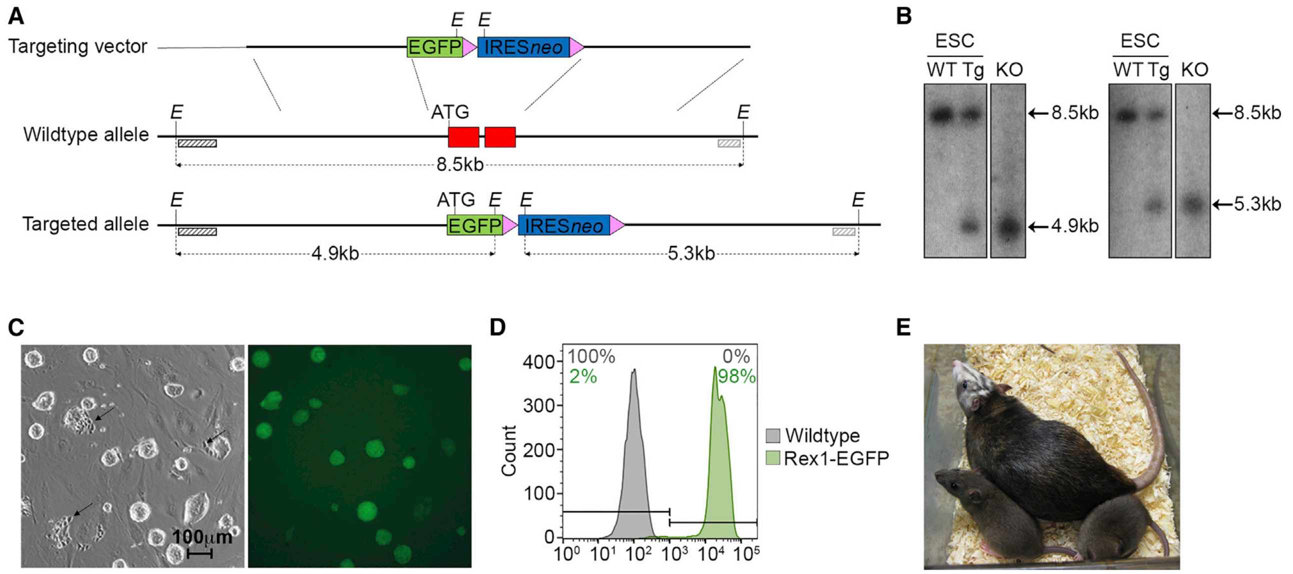

Figure 1. Generating a *Rex1*-EGFP Reporter in the Rat

(A) Structure of the *Rex1*-EGFP targeting vector (top), wild-type *Rex1* allele (middle), and targeted *Rex1* allele (bottom) resulting from replacement recombination at the dotted lines. The entire *Rex1* coding exon (red box) was replaced by a promoterless EGFP reporter (green box) and an IRES*neo* selection cassette (blue box with *LoxP* sites as pink arrows). Non-exonic chromosomal genomic DNA sequence is depicted by a thick black line and plasmid sequence by a thin black line. The restriction enzyme site *Eco*RI (*E*) and 5′ and 3′ probe sequences (dark and light hashed boxes respectively), external to the homology arms were used for Southern blot screening. The sizes of expected restriction digest fragments are shown by dotted arrows.

(B) Southern blot analysis of *Eco*RI-digested genomic DNA of wild-type (WT) ESCs, targeted ESCs (Tg), and *Rex1*-EGFP knockout (KO) rats using the 5′ and 3′ external probes (left and right panels respectively).

(C) Brightfield and fluorescent images of a *Rex1*-EGFP targeted clone (black arrows highlight morphologically differentiated *Rex1*-EGFP-negative cells, magnification ×100).

(D) Flow cytometry analysis of wild-type parental rat ESCs (gray shading) and the derivative *Rex1*-EGFP targeted clone (green shading).

(E) Photograph of *Rex1*-EGFP adult rat chimaera with agouti coat color ESC-derived offspring.

2014; Hosler et al., 1989; Kalkan et al., 2017; Rogers et al., 1991). The REX1 zinc finger protein arose through duplication of the YY1 transcription factor gene during radiation of eutherian mammals and is most highly expressed in the preimplantation embryo, within a specific region of the placenta, and in the testis (Kim et al., 2007; Rogers et al., 1991). It is reported to regulate X chromosome activity through induction of the antisense RNA Tsix that represses *Xist* expression (Navarro et al., 2010). REX1 may also function as an epigenetic regulator through association with Polycomb, and as a repressor of endogenous retroviruses or visceral endoderm-associated genes (Garcia-Tuñon et al., 2011; Guallar et al., 2012; Kim et al., 2011; Masui et al., 2008). Although there are indications that loss of REX1 may affect embryonic development and reduce fertility in aged mice, REX1-deficient mice are generally viable and healthy (Kalkan et al., 2017; Masui et al., 2008; Rezende et al., 2011). Indeed, in mouse ESCs the protein is dispensable for pluripotency and the *Rex1*-fluorescent protein knockin transgene is used as a sensitive reporter of pluripotency *in vitro* and as a tool to assess stem cell potential *in vivo* (Bhatia et al., 2013; Boroviak et al., 2014; Kalkan et al., 2017; Toyooka et al., 2008; Wray et al., 2011).

In this study we report the generation of a *Rex1*-EGFP knockin allele in the rat and examine expression of the *Rex1*-EGFP reporter during preimplantation embryo development and ESC derivation. We evaluate the general requirement for REX1 *in vitro* and *in vivo*, and characterize the utility of the *Rex1*-EGFP reporter for studying self-renewal and differentiation status of rESC cultures.

RESULTS

Generation of a *Rex1*-EGFP Reporter rESC and Rat

To assess the pluripotent state of rat ESCs accurately in live cultures we used conventional homologous recombination to insert a promoterless EGFP-IRES-neomycin resistance cassette immediately downstream of the *Rex1* (*Zfp42*) promoter, replacing the entire coding region of the *Rex1* gene (Figure 1A). Germline competent Dark Agouti

(DAK31) male rESCs (Blair et al., 2012) were electroporated with the linearized targeting vector, allowed to recover for 48 h, and then subjected to selection with the antibiotic G418 for a further 7 days. Ten G418-resistant ESC clones were expanded and all were shown by Southern blot analysis to carry the EGFP-IRES-neomycin cassette inserted within the *Rex1* gene (Figure 1B). Targeted clones displayed the typical rESC colony morphology and exhibited EGFP fluorescence as identified by fluorescence microscopy and flow cytometry (Figures 1C and 1D). qRT-PCR confirmed that *Rex1* mRNA levels were reduced by approximately 50% in the targeted *Rex1* heterozygous cells relative to wild-type parental cells (Figure S1).

The *Rex1*-EGFP clone E3 retained a normal karyotype and normal *in vitro* differentiation capacity. We also tested the developmental capacity of the E3 clone by assessing its ability to contribute to rat chimaeras following blastocyst injection. Clone E3 generated coat color chimaeras at a frequency of 41%, which was comparable with the 34% frequency obtained previously with the unmodified parental cell line, DAK31 (Table S1) (Meek et al., 2013). Seven male chimaeras were bred to test for ESC germline contribution, and two chimaeras fathered pups that demonstrated transmission of both coat-color and the *Rex1*-EGFP allele (Table S1 and Figure 1E).

To characterize the expression pattern of the *Rex1*-EGFP reporter *in vivo*, timed matings were used to generate *Rex1*-EGFP transgenic embryos. EGFP fluorescence was not detected in 1-, 2- and 4-cell embryos, but was evident in 8-cell (day E3.5) and blastocyst (day E4.5) stage embryos (Figure 2A). Interestingly, strong EGFP fluorescence was observed in both the inner cell mass (ICM) and the surrounding trophectoderm cells of the blastocyst, and persisted in the trophoblast cells after overnight culture, suggesting that *Rex1*-EGFP expression was probably not due to perdurance of EGFP protein from the earlier embryonic stages (Figure 2A). To assess the activity of the *Rex1*-EGFP reporter during ESC derivation, we plated heterozygous *Rex1*-EGFP E4.5 blastocysts into individual wells and monitored EGFP fluorescence during explant outgrowth for 7 days (Figure 2B). *Rex1*-dependent EGFP fluorescence was maintained throughout the expanding epiblast outgrowth. By contrast, EGFP fluorescence was gradually lost from the trophoblast cells and the surrounding flattened differentiated cells. After 7 days, the ICM outgrowths were dissociated and replated to produce heterozygous *Rex1*-EGFP ESC lines that were generated at a frequency comparable with wild-type ESC lines (Figure S2).

To assess the functional requirement for REX1 in rats we genotyped the offspring from multiple crosses between *Rex1*-EGFP heterozygous rats. Litters displayed a near-normal Mendelian ratio of wild-type (20%), *Rex1*-EGFP heterozygous (60%), and *Rex1*-EGFP homozygous (20%) pups (Table S2). Moreover, the behavior and general health of the *Rex1*-EGFP homozygous rats was indistinguishable from wild-type or heterozygous littermates. Previous reports have suggested that REX1-deficient male mice are sub-fertile (Rezende et al., 2011). We therefore compared the litter sizes of *Rex1*-EGFP homozygous males with age-matched wild-type control males (9–12 months old) but could not detect any obvious difference in average litter numbers at E4.5 or mid-gestation between homozygous and wild-type rats, suggesting that the fertility of *Rex1* mutant male rats at the ages we tested was normal (Table S3).

We next investigated how disruption of *Rex1* affected the derivation and maintenance of rESCs *in vitro*. The culture of E4.5 embryos generated by cross-breeding heterozygous *Rex1*-EGFP rats showed that the derivation efficiency of *Rex1*-EGFP homozygous ESCs under 2i+LIF conditions (83%) was close to that obtained for wild-type (100%) and heterozygous (91%) lines (Figure S2). Furthermore, *Rex1*-EGFP homozygous ESC colonies were morphologically indistinguishable from those of *Rex1*-EGFP heterozygous or wild-type cells (Figure 2C). Notably, gene expression analysis by qRT-PCR confirmed the absence of *Rex1* expression in homozygous lines, but most significantly also demonstrated the absence of *Tsix* expression, a downstream target of REX1 (Navarro et al., 2010), thus confirming the loss of REX1 function (Figure 2D). Importantly, we did not detect consistent differences between clones in the expression of other key ESC genes (Figure 2E). In summary, we were unable to identify any consistent identifiable phenotype associated with the disruption of *Rex1* in the rat or ESCs. These findings indicate that the rat *Rex1* gene function is largely redundant in the rat and ESCs under standard conditions, and as a result provides a convenient neutral locus suitable for harboring a fluorescent reporter for monitoring stem cell pluripotency and self-renewal.

Rex1-EGFP Reporter Expression Discriminates between Pluripotent and Differentiated ESC States

To evaluate the specificity of the rat *Rex1*-EGFP reporter, we monitored the correspondence between expression of *Rex1* mRNA and *Rex1*-EGFP mRNA under culture conditions that promote ESC differentiation. Withdrawal of LIF and 2i inhibitors from rESCs in suspension cultures to promote embryoid body differentiation reduced the level of EGFP expression as assessed by fluorescence microscopy (Figure 3A). qRT-PCR confirmed coincident downregulation of EGFP and endogenous *Rex1* mRNAs, as well as reduced expression of the pluripotency-associated gene *Nanog* in embryoid bodies (Figure 3B). Similarly, a reduction in the concentration of MEK inhibitor applied to the monolayer

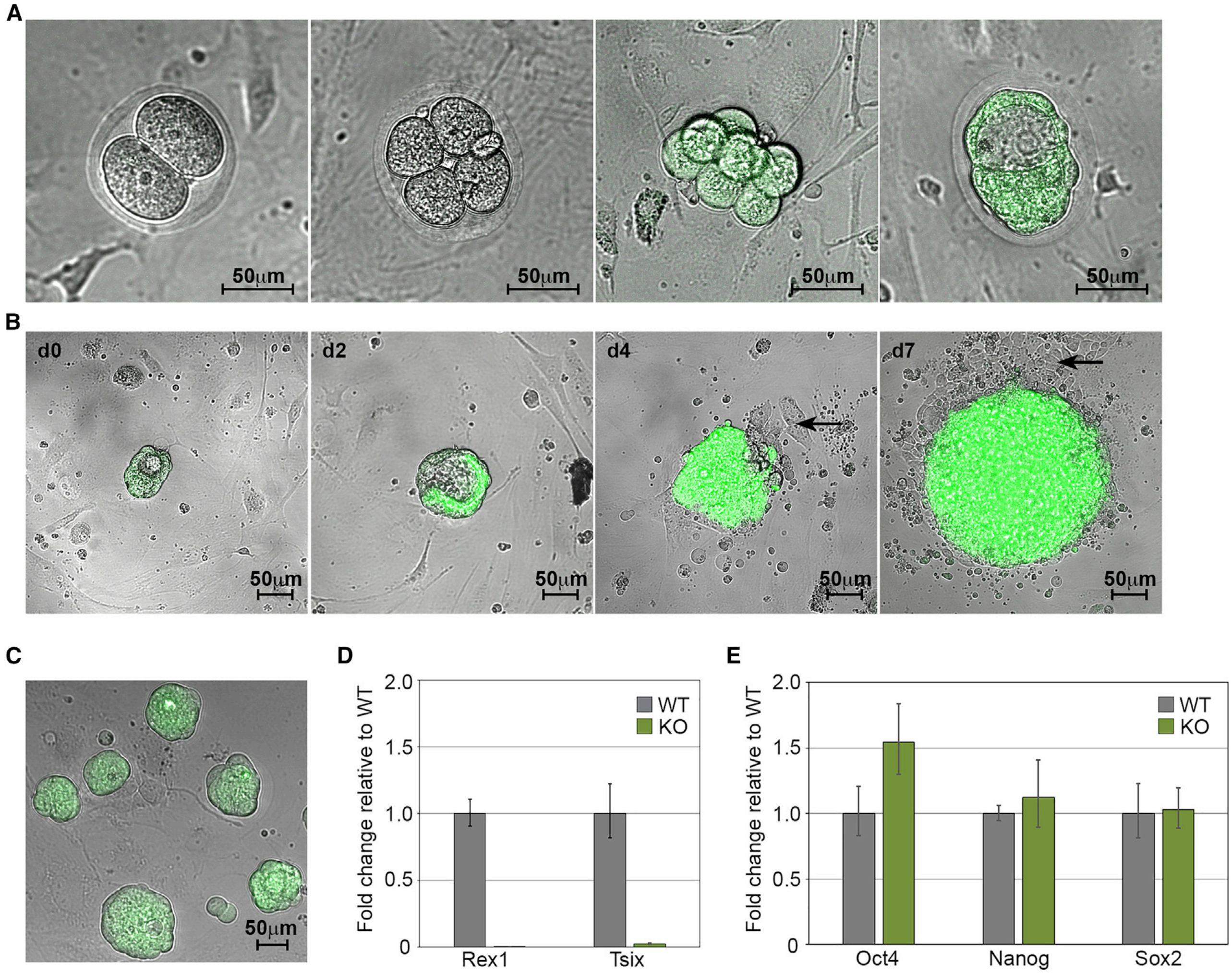

Figure 2. *In Vivo* and *In Vitro Rex1*-EGFP expression pattern

(A and B) Compound brightfield and fluorescent images of *Rex1*-EGFP expression (A) in 2-cell, 4-cell, 8-cell, and blastocyst-stage rat embryos (in sequence from left to right), and (B) during ESC derivation in a day 0 (d0), d2, d4, and d7 outgrowth from an E4.5-derived rat blastocyst (arrows highlight *Rex1*-EGFP-negative extraembryonic cells).

(C) Compound brightfield and fluorescent image of *Rex1*-EGFP KO rat ESCs (magnification ×100).

(D) qRT-PCR analysis for *Rex1* and *Tsix* expression in wild-type (WT) and knockout (KO) rat ESC (mean ± sd of three biological replicates).

(E) qRT-PCR analysis of the core pluripotency transcription factors *Oct4*, *Nanog* and *Sox2* in wild-type (WT) and knockout (KO) rat ESC (mean ± sd of three biological replicates).

cultures produced a dose-dependent decline in EGFP-positive cells within the culture that was accompanied by a coordinate reduction in *Rex1* and *Rex1*-EGFP mRNA expression, and increased expression of an early differentiation marker *Gata4* (Figures 3C and 3D).

High levels of β-catenin (CTNNB1) activity in association with the transcription factor LEF1 drives rat ESC differentiation, causing a loss of ESC colony morphology, induction of mesendoderm-associated differentiation markers Brachyury and Cdx2, and increased instability and collapse of rESC cultures (Chen et al., 2013; Meek et al., 2013). We therefore examined the response of the *Rex1*-EGFP reporter to manipulation of the β-catenin/LEF1 signaling pathway in rESCs. Fluorescence microscopy and flow cytometry confirmed that induction of β-catenin activity using concentrations of the GSK inhibitor CHIR99021 (CH) >3 μM efficiently suppressed *Rex1*-EGFP-dependent fluorescence (Figures 4A and 4B) and induced ESC differentiation (Figure 4C). Depletion of β-catenin (Ctnnb1) or LEF1 in these culture conditions, by either small interfering RNA (siRNA)-mediated knockdown (Figures S3A and S3B) or TALEN-mediated gene editing, maintained *Rex1*-EGFP

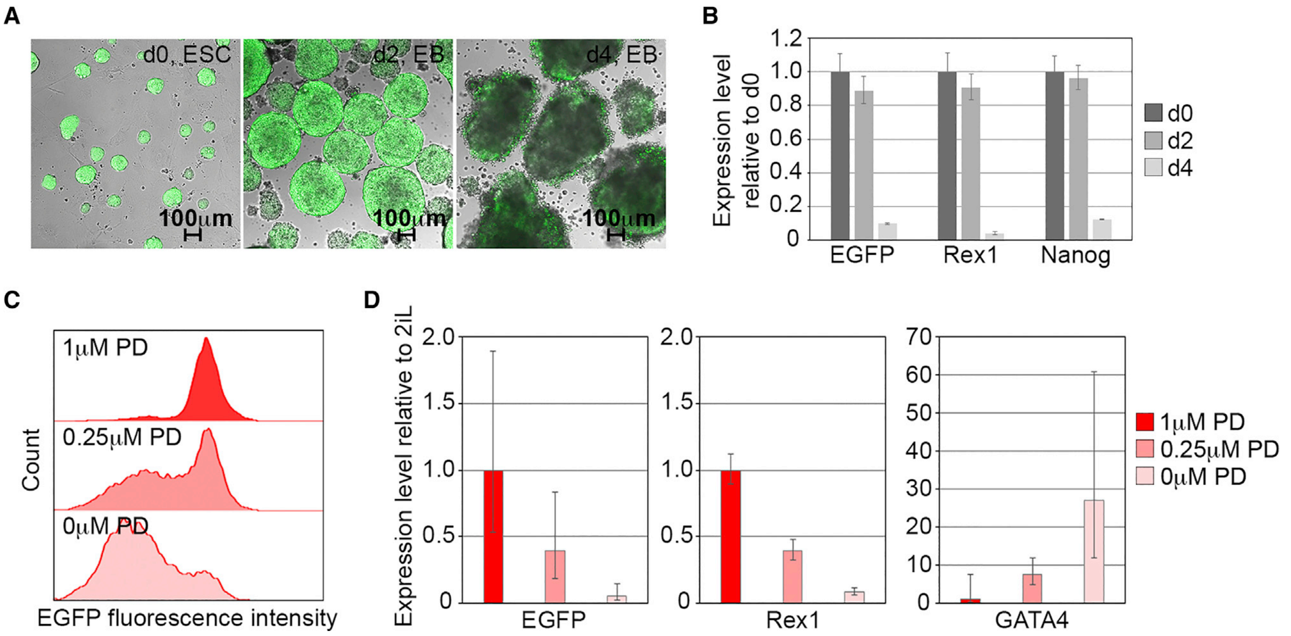

Figure 3. Downregulation of *Rex1*-EGFP Reporter Expression during *In Vitro* Differentiation

(A) Compound brightfield and fluorescent images of *Rex1*-EGFP expression during embryoid body formation at d0 (left panel), d2 (middle panel), and d4 (right panel).

(B) qRT-PCR analysis of EGFP, *Rex1*, and *Nanog* expression in *Rex1*-EGFP rat ESCs harvested at d0, d2, and d4 during embryoid body differentiation (mean ± SD of three technical replicates).

(C) Flow cytometry analysis of *Rex1*-EGFP rat ESCs cultured for four days in 3 μM CHIR99021+LIF containing 1 μM PD0325901 (top panel), 0.25 μM PD (middle panel), and without PD (bottom panel).

(D) qRT-PCR analysis of EGFP, *Rex1*, and *Gata4* in *Rex1*-EGFP rat ESCs cultured for four days in 3 μM CHIR99021+LIF containing 1 μM PD, 0.25 μM PD, and without PD (mean ± SD of three biological replicates).

expression and ESC self-renewal (Figures 4C and 4D). Sequence analysis of β-catenin and LEF1 TALEN-transfected clones confirmed the presence of inactivating mutations in the *Ctnnb1* and *Lef1* alleles, respectively (Figure S3C). Transfection with control siRNAs or control TALENs, in contrast, did not prevent CH-induced rESC differentiation or downregulation of *Rex1*-EGFP reporter expression.

To determine if the *Rex1*-reporter could also be reactivated upon dedifferentiation into a pluripotent stem cell state through induced pluripotent stem cell (iPSC) reprogramming, we transfected rat embryonic fibroblasts (REFs) derived from a *Rex1*-EGFP heterozygous embryo with a piggyBac vector co-expressing human cDNAs encoding OCT4, SOX2, KLF4, and cMYC (Gao et al., 2019). The starting REF cultures did not express EGFP but 7–10 days after transfection with the piggyBac construct, compact colonies typical of undifferentiated rESCs emerged within the cultures that had reactivated the *Rex1*-EGFP reporter and expression of the endogenous rESC transcription factor NANOG (Figures 4E and S3D). This confirmed that the *Rex1*-EGFP reporter can be used to monitor iPSC reprogramming and together with rESC differentiation data indicates that the rat *Rex1*-EGFP allele provides a useful marker of undifferentiated pluripotent rat stem cells and experimentally manipulated derivatives.

Rex1-EGPF Reporter Expression Reveals Functional Heterogeneity in Rat ESC Cultures Maintained in 2i+LIF Conditions

Rat ESCs propagated in 2i+LIF culture medium exhibit background levels of spontaneous differentiation (Blair et al., 2012). To assess how the *Rex1*-EGFP reporter could be used to monitor the pluripotent status of cells within rESC cultures, we analyzed expression of the reporter in rat ESCs grown under standard 2i+LIF growth conditions with feeder support. Under these circumstances the basal level of spontaneous differentiation is most evident as morphologically differentiated cells located at the periphery of some ESC colonies. Immunocytochemistry revealed that many of these cells expressed the primitive endoderm-associated transcription factor GATA4 (Figure 5A). Expression of the *Rex1*-EGFP reporter showed the opposite pattern, being restricted to undifferentiated cells and excluded from the peripheral differentiated cells (Figure 5A). GATA4$^+$/*Rex1*-EGFP$^-$ cells were also evident within

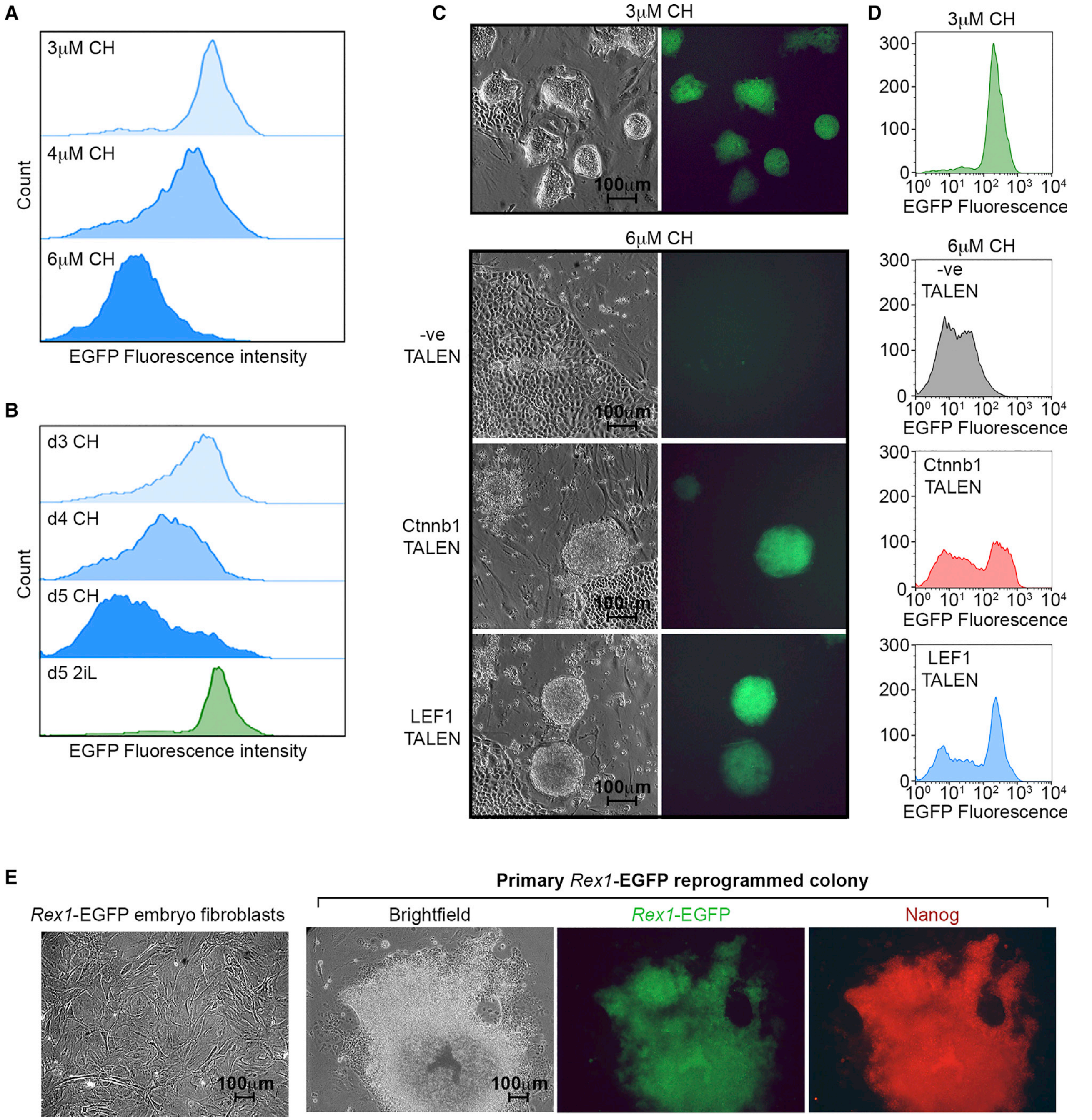

Figure 4. Response of *Rex1*-EGFP Reporter to CHIR-Induced Differentiation and Rescue by Targeting β-Catenin and LEF1

(A) Flow cytometry analysis of *Rex1*-EGFP rat ESCs cultured for five days in 2i medium containing 3 μM, 4 μM, or 6 μM CHIR99021.

(B) Flow cytometry analysis of *Rex1*-EGFP rat ESCs cultured for 3, 4, or 5 days in 2i medium containing 6 μM CHIR99021, and 5 days in 2iL.

(C) Brightfield and fluorescent images of *Rex1*-EGFP rat ESCs cultured for four days in 2iL self-renewal (3 μM CHIR99021) and differentiating (6 μM CHIR99021) rat ESC culture conditions, following transfection with control (-ve), *Ctnnb1*, or *Lef1*-specific TALENs (magnification ×100).

(D) Flow cytometry analysis of *Rex1*-EGFP rat ESCs cultured for four days in conditions described in (C).

(E) Brightfield image of *Rex1*-EGFP REFs, and brightfield, fluorescent (EGFP), and immunostained (Nanog) images of a *Rex1*-EGFP rat iPSC colony (magnification ×100).

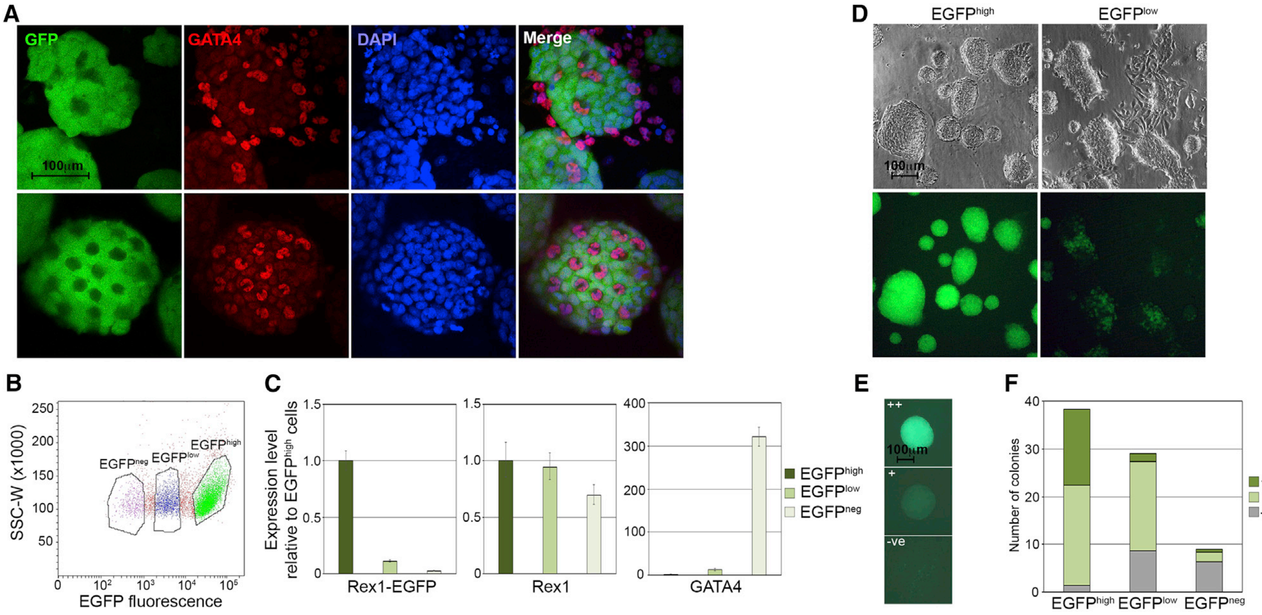

Figure 5. *Rex1*-EGFP Reveals Functional Heterogeneity in 2iL Rat ESC Cultures

(A) Fluorescence and immunocytochemistry of examples of 2iL *Rex1*-EGFP rat ESC colonies imaged by confocal microscopy for EGFP and GATA4 expression respectively. DAPI-stained cells and merged images are also shown (magnification ×400).

(B) Flow cytometry analysis of 2iL *Rex1*-EGFP rat ESCs, showing EGFP^{neg}, EGFP^{low}, and EGFP^{high} populations.

(C) qRT-PCR analysis of *Rex1*-EGFP, *Rex1*, and *Gata4* expression in fluorescence-activated cell (FAC)-sorted *Rex1*-EGFP^{high}, *Rex1*-EGFP^{low}, and *Rex1*-EGFP^{neg} rat ESCs (mean ± SD of three technical replicates).

(D) Brightfield and fluorescent images of rat ESC cultures five days after replating FAC-sorted *Rex1*-EGFP^{high} and *Rex1*-EGFP^{low} cells using gates applied in (B).

(E) Representative fluorescent images of colonies scored as EGFP^{high}, EGFP^{low}, and EGFP^{neg} seven days after plating single cells FAC sorted using gates applied in (B).

(F) Quantitation of EGFP^{high}, EGFP^{low}, and EGFP^{neg} rat ESC colonies formed seven days after plating FAC-sorted *Rex1*-EGFP^{high}, *Rex1*-EGFP^{low}, and *Rex1*-EGFP^{neg} single cells.

some of the otherwise morphologically undifferentiated ESC colonies, implying that differentiation could also occur within the interior of the colonies.

We performed flow cytometry on the bulk cultures to quantify *Rex1*-EGFP expression in standard rESC 2i+LIF cultures and found that the ESCs could be assigned to three populations: cells expressing high levels of EGFP (*Rex1*-EGFP^{high}), which typically represented the majority (>90%) of cells in the culture; cells expressing an intermediate or low level of EGFP (*Rex1*-EGFP^{low}), which constituted a variable and less distinct population (1–10%); and cells that did not express EGFP (*Rex1*-EGFP^{neg}) and contributed to 1–3% of the overall population (Figure 5B). qRT-PCR of the flow-sorted populations showed that *Rex1*-EGFP mRNA expression was downregulated in the *Rex1*-EGFP^{low} cells, and almost absent from the *Rex1*-EGFP^{neg} population (Figure 5C). By contrast, expression of the differentiation marker Gata4 was absent from the *Rex1*-EGFP^{high} population, but was upregulated stepwise in *Rex1*-EGFP^{low} and *Rex1*-EGFP^{neg} cells, confirming the progressively increasingly differentiated status of cells within these populations. Expression of endogenous *Rex1* mRNA was downregulated in *Rex1*-EGFP^{neg} cells but unexpectedly was expressed at high levels in the *Rex1*-EGFP^{low} population. This suggested that *Rex1*-EGFP^{low} cells represented an intermediate state between the undifferentiated *Rex1*-EGFP^{high} and differentiated *Rex1*-EGFP^{neg} cells, characterized by asynchronous downregulation of the *Rex1* alleles. This asynchronous pattern of downregulation of targeted and non-targeted *Rex1* alleles was confirmed in three additional, independently targeted, *Rex1*-EGFP ESC clones (Figure S4).

We next examined the stem cell self-renewal potential of cells within the *Rex1*-EGFP sub-populations, by purifying *Rex1*-EGFP^{low} and *Rex1*-EGFP^{high} cells by fluorescence-activated cell sorting (FACS), replating the cells in 2i+LIF ESC self-renewal conditions, and monitoring the growth of the resulting cultures for 4 days (Figure 5D). Most *Rex1*-EGFP^{high} cells formed EGFP-positive, compact, spherical colonies typical of undifferentiated rESC colonies. By

contrast, *Rex1*-EGFP^{low} cells formed more irregularly shaped and flattened colonies containing cells expressing variable levels of EGFP. Many of these colonies were also surrounded by flat, morphologically differentiated cells (Figure 5D). To stringently assess the self-renewal potential of individual rat ESCs within the EGFP sub-populations, we sorted cells according to their levels of EGFP expression (high, low, and negative), and assessed the colony-forming potential of the cells in a single-cell cloning assay. Individual cells were deposited into 96-well plates and after 7 days of culture the resulting colonies were scored for EGFP fluorescence and morphology (Figures 5E and 5F). *Rex1*-EGFP^{high} cells exhibited the highest colony-forming potential and generated undifferentiated EGFP^{high} colonies at the highest frequency (42%). A significant proportion of the EGFP^{high} cells (55%) also produced colonies that expressed uniformly low levels of EGFP. *Rex1*-EGFP^{low} sorted cells gave rise to fully differentiated *Rex1*-EGFP^{neg} colonies (31%), but also produced morphologically undifferentiated colonies that maintained a low level of EGFP expression (65%), indicating that the *Rex1*-EGFP^{low} state could be maintained through multiple cell divisions. By comparison, *Rex1*-EGFP^{neg} cells exhibited reduced cloning efficiency, gave rise to some *Rex1*-EGFP^{low} colonies (22%) and produced the greatest proportion of morphologically differentiated *Rex1*-EGFP-negative colonies (67%). The clonal analysis demonstrated that expression of the *Rex1*-EGFP reporter could discriminate between different rat ESC states. It also identified a loss of pluripotent potential among the *Rex1*-EGFP^{low} population and indicated that this state could persist for a period of time under standard 2i+LIF culture conditions. To assess how this loss of potential affected developmental potency *in vivo*, we purified *Rex1*-EGFP^{low} cells by FACS, allowed them to recover in culture for 4 days, and tested their contribution to embryonic development by blastocyst injection. The cells contributed to live-born chimaeras with an efficiency close to the parental ESCs (6 chimaeras out of 26 pups: 23%), demonstrating that the *Rex1*-EGFP^{low} cells either fully retained the capacity to functionally reintegrate into the developing embryo or were in a state where this potential was readily reinstated within the blastocyst environment.

To examine the gene expression profile of the *Rex1*-EGFP^{low} intermediate cell type, we assessed the transcriptional activity of cells within the three populations of *Rex1*-EGFP cells in 2i+LIF culture by single-cell RNA sequencing. We sorted cells into replicate 96-well plates according to their levels of EGFP expression and followed the Smart-seq2 protocol, with minor modifications as we have described previously (Kirschner et al., 2017; Picelli et al., 2014) (Figure 6A). Consistent with the sorting criteria, the average level of EGFP RNA decreased in the populations in the order EGFP^{high}>EGFP^{low}>EGFP^{neg} (Figure 6B). Conversely, expression of the differentiation markers Gata4 and Sox17 was upregulated in the EGFP^{neg} population. Expression of the ESC pluripotency-associated factors *Nanog* and *Klf2* was also downregulated in the *Rex1*-EGFP^{neg} cells (Figure 6B). Most ESC factors, however, were expressed at comparable levels in the *Rex1*-EGFP^{high} and *Rex1*-EGFP^{low} cells, indicating that *Rex1*-EGFP^{low} cells might represent a very early step in the process of exiting pluripotency. Indeed, *Rex1* mRNA expression from the non-targeted *Rex1* allele in the *Rex1*-EGFP^{low} cells was at a similar or higher level than in *Rex1*-EGFP^{high} cells, even though *Rex1* mRNA was clearly downregulated in the differentiating *Rex1*-EGFP^{neg} population (Figure 6B). This differential downregulation of wild-type *Rex1* mRNA and *Rex1*-EGFP mRNA in the *Rex1*-EGFP^{low} cells is consistent with bulk analysis of the rESC population (Figures 5C and S4) and supports the notion that asynchronous downregulation of the *Rex1* alleles identifies an intermediary rESC state. The direct comparison of EGFP mRNA and *Rex1* mRNA expression in individual cells from all three populations showed that within many *Rex1*-EGFP^{high} (*Rex1*$^{mRNA+/EGFP+}$) and *Rex1*-EGFP^{neg} (*Rex1*$^{mRNA-/EGFP-}$) cells, both *Rex1* alleles were active or inactive, respectively (Figure 6C). By contrast, the majority of the cells within the *Rex1*-EGFP^{low} population expressed high levels of *Rex1* mRNA (Rex1$^{mRNA+/EGFP-}$). To assess the differentiation potential of *Rex1*-EGFP^{low} cells *in vitro*, we purified the cells by FACS, expanded the population for 4 days after plating, and then analyzed the cells by flow cytometry and single-cell sequencing. In line with previous results, a significant proportion of the cells retained a *Rex1*-EGFP^{low} state (Figure 6D). In addition, expression of *Rex1* mRNA was downregulated in many of the remaining cells and the overall transcriptional profile of the cells shifted away from the pre-plated *Rex1*-EGFP^{low} and *Rex1*-EGFP^{high} cells, consistent with many of the cells having differentiated (Figures 6E and 6F).

Collectively these results suggest that the *Rex1*-EGFP reporter reveals a background level of differentiation in rESC 2i+LIF cultures that proceeds in the following order: from *Rex1*$^{mRNA+/EGFP+}$ → *Rex1*$^{mRNA+/EGFP-}$ → *Rex1*$^{mRNA-/EGFP-}$. To investigate this possibility further we specifically compared the transcription profiles of stem cell pluripotency regulator genes in cells within these three categories (Figures 6G and 6H). This analysis revealed a trend of downregulation of pluripotency-associated factors in the *Rex1*$^{mRNA+/EGFP-}$ cells compared with *Rex1*$^{mRNA+/EGFP+}$ cells, some of which, such as *Tbx3*, *Klf2*, *Klf4*, and *Klf5*, are commonly associated with naive pluripotency (Figures 6I and S5). Gene list enrichment analysis (ToppGene Suite) showed enrichment of genes involved in chromosome organization among those differentially regulated between *Rex1*$^{mRNA+/EGFP-}$ and *Rex1*$^{mRNA+/EGFP+}$ cells (Figure S6).

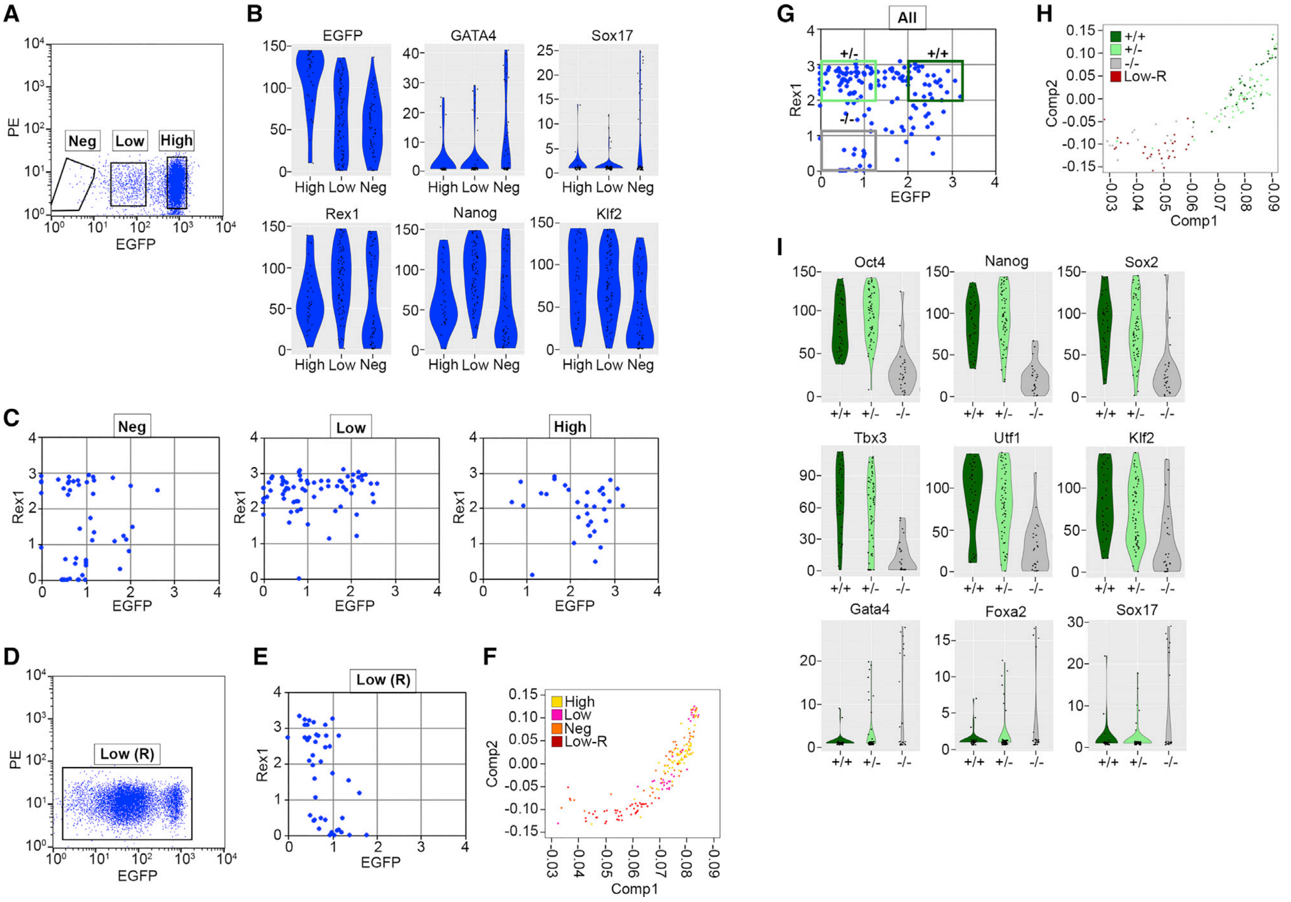

Figure 6. Single-Cell RNA Sequence Analysis of Heterogeneity in 2iL Rat ESC Cultures

(A) Flow cytometry analysis of 2iL *Rex1*-EGFP rat ESCs, showing the gates used to sort the *Rex1*-EGFP^{neg}, *Rex1*-EGFP^{low}, and *Rex1*-EGFP^{high} populations.

(B) Violin plots showing the expression levels of ESCs and differentiation markers in the *Rex1*-EGFP^{high}, *Rex1*-EGFP^{low}, and *Rex1*-EGFP^{neg} cell populations derived from the single-cell analysis.

(C) Expression of *Rex1* mRNA and *Rex1*-EGFP mRNA in *Rex1*-EGFP^{high}, *Rex1*-EGFP^{low}, and *Rex1*-EGFP^{neg} single cells.

(D) Flow cytometry showing EGFP expression in *Rex1*-EGFP^{low} cells 4 days after replating.

(E) Expression of *Rex1* mRNA and *Rex1*-EGFP mRNA in replated *Rex1*-EGFP^{low} cells.

(F) PCA analysis of gene expression in *Rex1*-EGFP^{high}, *Rex1*-EGFP^{low}, Rex1-EGFP^{neg}, and *Rex1*-EGFP^{low} replated cells.

(G) Expression of *Rex1* mRNA and *Rex1*-EGFP mRNA in single cells, with the gates used to select *Rex1*$^{mRNA+/EGFP+}$, *Rex1*$^{mRNA+/EGFP-}$, and *Rex1*$^{mRNA-/EGFP-}$ cells highlighted.

(H) PCA analysis of gene expression in *Rex1*$^{mRNA+/EGFP+}$, *Rex1*$^{mRNA+/EGFP-}$, *Rex1*$^{mRNA-/EGFP-}$, and *Rex1*-EGFP^{low} replated cells.

(I) Violin plots of pluripotency and differentiation marker expression in *Rex1*$^{mRNA+/EGFP+}$, *Rex1*$^{mRNA+/EGFP-}$, and *Rex1*$^{mRNA-/EGFP-}$ cells.

In summary, these results show that the *Rex1*-EGFP reporter can be used to distinguish between pluripotent rat ESC states and their differentiated derivatives, and provides a useful tool for exploring early stages during establishment and dissolution of ESC pluripotency.

DISCUSSION

Rex1 (*Zfp42*) gene expression is a benchmark marker of pluripotency in cultures of ESCs and reprogrammed iPSCs (Bao et al., 2011; Bhatia et al., 2013; Kalkan et al., 2017; Rodríguez et al., 2012). Here we describe the characterization of a *Rex1*-EGFP knockin transgene in the rat, and show that it provides a sensitive reporter of the pluripotent state in rESCs. Rat ESCs differ somewhat from mouse ESC in their growth factor requirements and growth characteristics, thereby providing a unique alternative to mouse cells with which to investigate self-renewal signaling and pluripotency (Chen et al., 2013; Li et al., 2008; Meek et al., 2013). Based on the work presented here we propose that the *Rex1*-EGFP reporter provides a useful tool to interrogate

the early stages of ESC differentiation in the rat, and enrich our understanding of the regulation of pluripotency in mammals.

Replacement of the *Rex1* open reading frame with the EGFP cassette eliminated *Rex1* expression and function. Nonetheless, disruption of *Rex1* expression did not adversely affect either the derivation or growth of rESCs, or apparently the health of *Rex1* knockout rats. This is in line with previous reports showing that *Rex1* function was dispensable for maintaining pluripotency in mouse ESCs, and not essential in mice (Masui et al., 2008). However, in contrast to reports in mice, we did not observe signs of selective loss of *Rex1* homozygous mutant embryos or a loss of fertility in male knockout rats (Kalkan et al., 2017; Rezende et al., 2011), nor observe a bias toward endoderm differentiation previously described for mouse *Rex1* knockout ESCs (Masui et al., 2008). Nonetheless, deletion of the *Rex1* coding region in rat ESCs did eliminate expression of *Tsix*, a non-coding RNA that suppresses X chromosome inactivation and a reported target of REX1 in mouse (Navarro et al., 2010). This result confirmed that the *Rex1*-EGFP knockin created a non-functional allele and pointed to a conserved role of *Rex1* in epigenetic regulation of X chromosome activity in rat and mouse. Taken together our results indicate that REX1 function is largely dispensable, and implies that the *Rex1*-EGFP knockin allele provides a neutral reporter that does not noticeably interfere with ESC self-renewal or normal embryonic development in the rat.

Embryonic expression of the *Rex1*-EGFP reporter was first detected at the 8-cell stage and thereafter persisted through compaction to the formation of the blastocyst. *Rex1*-EGFP was detected within the external trophoblast cells of the blastocyst as well as the ICM, implying that early expression *in vivo* in the rat embryo is associated with an early undifferentiated embryonic state, rather than exclusively as a marker of pluripotency. Indeed, *Rex1* expression has been detected in trophoblast cell types in the mouse, including cells within the ectoplacental cone, extraembryonic ectoderm, and the placenta of post-implantation embryos (Kalkan et al., 2017; Kim et al., 2011; Rogers et al., 1991). In the mouse blastocyst, however, *Rex1* or *Rex1*-EGFP mRNA expression was restricted to the ICM, implying that the level or onset of *Rex1* expression in the trophoblast lineage differs between the mouse and the rat embryos (Kalkan et al., 2017; Pelton et al., 2002). Nevertheless, when rat blastocysts were cultured in 2i+LIF ESC culture conditions, *Rex1*-EGFP expression became rapidly confined to the expanding pluripotent stem cell compartment, consistent with *Rex1*-EGFP expression marking the undifferentiated ESCs. This restricted pattern of *Rex1*-EGFP expression might be further sharpened by failure of trophoblast cells to thrive in the 2i culture conditions (Ying et al., 2008). Withdrawal of self-renewal conditions or active stimulation of differentiation, by contrast, resulted in downregulation of the *Rex1*-EGFP reporter along with the unmodified wild-type *Rex1* allele and other markers of ESC pluripotency.

Even under standard 2i+LIF culture conditions, the activity of the *Rex1*-EGFP reporter gene could discriminate between undifferentiated ESCs and cells spontaneously escaping pluripotency and embarking on differentiation. An early transition state was identified by a low-level, semi-stable expression of the *Rex1*-EGFP reporter and was functionally characterized by an increased tendency to differentiate under clonal growth conditions. Surprisingly, expression of the untargeted *Rex1* allele in these transitional cells was as high as in rat ESCs expressing high levels of *Rex1*-EGFP (*Rex1*-EGFP^{high}). This pointed to selective loss of transcriptional activity from the targeted *Rex1*-EGFP allele and might arise from a loss of positive regulatory elements from the targeted *Rex1*-EGFP allele or interference induced by insertion of the targeting cassette, thus sensitizing expression of the EGFP knockin allele to a weakening of control of pluripotency. An alternative possibility, however, is that asynchronous expression of *Rex1* alleles might occur normally in this peri-naive ESC transition state. Indeed, the detection of *Rex1*-EGFP^{high} cells expressing low levels of endogenous *Rex1* mRNA (*Rex1*$^{mRNA-/EGFP+}$), thus mirroring the *Rex1*-EGFP^{low} (*Rex1*$^{mRNA+/EGFP-}$) population, supports the notion that the EGFP transgene might simply reflect asynchrony in the response of *Rex1* alleles to relaxation of the control of pluripotency in the earliest stages of differentiation. Indeed, the pluripotency factor *Nanog* has been reported to exhibit mono-allelic expression prior to attaining full biallelic expression in the ICM of the blastocyst (Miyanari and Torres-Padilla, 2012).

Notwithstanding the asynchronous downregulation of the Rex1 alleles, the reduction in EGFP expression in *Rex1*-EGFP^{low} cells potentially identified an intermediary rESC state at a very early stage in differentiation. This notion was supported by the finding that cells expressing low levels of *Rex1*-EGFP mRNA while retaining high levels of *Rex1* mRNA (*Rex1*$^{mRNA+/EGFP-}$) also tended to express lower levels of naive pluripotency regulators (Nichols and Smith, 2009) compared with the *Rex1*-EGFP^{high} (*Rex1*$^{mRNA+/EGFP+}$) cells. Pathway analysis of genes differentially regulated between these two populations also pointed to the involvement of chromosome reorganization during this transition. Interestingly, we did not observe significant levels of reversion of the *Rex1*-EGFP^{low} cells to the *Rex1*-EGFP^{high} state in single-cell clonal assays, in line with the results reported for mouse *Rex1*-EGFP^{low} ESCs cultured in 2i+LIF conditions (Kalkan et al., 2017). This contrasts with the results reported for a *Rex1*-EGFP reporter in mouse ESCs propagated in serum+LIF medium

(Toyooka et al., 2008) and suggests that the stringency associated with 2i+LIF clonal culture conditions provides an effective barrier for the reversion of *Rex1*-EGFP^{low} cells to a *Rex1*-EGFP^{high} state. In conjunction with the persistence of the intermediate state in rat ESCs, this might potentially provide a continuous source of instability in rat ESC cultures. Nonetheless, given that the progeny of *Rex1*-EGFP^{low} cells did colonize blastocysts and contribute to chimeric rats, it remains possible that, under the appropriate modified culture conditions, *Rex1*-EGFP^{low} cells might be induced to revert and be stably retained within a *Rex1*-EGFP^{high} state.

In conclusion, we show here that the rat *Rex1*-EGFP fluorescent reporter allows the qualitative and quantitative analysis of ESC states in rat ESC cultures *in vitro*, and provides a useful and sensitive tool to scrutinize early stages of pluripotent stem cell differentiation in the rat, as well as contributing to more general insights into the regulation of stem cell potency and fate determination in mammals.

EXPERIMENTAL PROCEDURES

ESC Culture

ESCs were derived from E4.5 rat blastocysts following removal of the zona pellucida with acidic Tyrode solution. The intact blastocyst was then cultured in the well of a 96-well plate, in 2iL (N2B27 medium, 1 μM PD0325901 [PD], 3 μM CHIR99021 [CH], 1000 U/mL mouse LIF) on γ-irradiated (5 Gy) OF1 mouse fibroblasts for 7 days prior to passaging. ESCs were maintained on irradiated OF1 or DR4 mouse fibroblasts in 2iL. Colonies were passaged every 2–3 days using TVP (0.025% trypsin, 1% chicken serum, and 1 mM EDTA) and plated at a density of (0.5–1) × 10^5/cm^2. MEK (PD) and GSK3 (CH) inhibitors were supplied by Axon Medchem (http://www.axonmedchem.com).

Gene Targeting by Homologous Recombination

A targeting vector was constructed using BAC recombineering which consisted of approximately 3 kb and 2.7 kb, 5′ and 3′ homology arms respectively, in which the entire *Rex1* coding sequence was replaced with a Kozak-EGFP-*lox*P-IRES*neo*-*lox*P-bGHpA reporter/selection cassette. Approximately 1 × 10^7 DAK31 rat ESCs (Blair et al., 2012) in 0.6 mL of PBS containing 100 μg of linearized *Rex1*-EGFP targeting vector were electroporated using a Bio-Rad Genepulser apparatus (0.8 kV, 3 μF). Electroporated cells were plated into 10 cm^2 wells containing 2iL medium. The aminoglycoside G418 (80 μg/mL) was added 48 h after electroporation and G418-resistant colonies picked 9 days later onto DR4 fibroblasts in 2iL in wells of a 96-well plate and expanded.

ESC Differentiation

For embryoid body differentiation, a single-cell suspension of rat ESCs was plated into non-coated, low-adherence plastic wells of a 6-well plate at a density of 2 × 10^5/cm^2 in 2iL. The cells were cultured for 2 days then transferred into fibroblast medium (Glasgow minimum essential medium [GMEM], 10% fetal calf serum [FCS]) for a further 2 days. Rat ESCs differentiated by culturing at a density of 5 × 10^3/cm^2 in either 2iL with concentrations of PD lower than 1 μM for 3–4 days or in 2i (no LIF) with concentrations of CH >3 μM for 3–4 days.

Reprogramming of REFs

Approximately 5 × 10^5 *Rex1*-EGFP REFs were resuspended in Resuspension Buffer R (Invitrogen) and transfected with 1.5 μg of pPBTRE-hOSMK + 0.5 μg of PBase + 0.5μg of PB-rTTA (Gao et al., 2019), using the Neon transfection system (Invitrogen) set at 1350 V, 30 ms, 1 pulse. Transfected cells were plated in a 6-well plate at a density of 2.4 × 10^3/cm^2 on γ-irradiated (100 Gy) STO mouse fibroblasts in M15G+SB medium (GMEM base media + 15% fetal bovine serum + 1000 U/mL human LIF + 50 μg/mL Vc + 1 μg/mL doxycycline + 1 mM sodium butyrate) and kept at 37°C in 5% CO_2. The medium was changed the day after transfection and every 2 days thereafter. Colonies emerged by day 10 and M15G+SB media changed to t2i+Lif (N2B27 medium, 1 mM PD0325901, 1 mM CHIR99021, 1000 U/mL mouse LIF). The colonies were fixed for immunocytochemistry at day 14.

Immunocytochemistry

Cells were fixed in cold 100% methanol (5 min at −20°C), washed with PBS, then blocked in PBS + 10% FCS for 1 h at room temperature. GATA4 primary antibody (Santa Cruz, sc-25310, 1:50) diluted in PBS/2%FCS was applied at room temperature for 2 h, followed by washes with PBS. Secondary antibody (goat anti-mouse IgG2a, 1:1000) was diluted in PBS/2%FCS and applied for 1 h at room temperature in the dark. The cells were washed with PBS, then 10 μg/mL DAPI applied for 5 min at room temperature in the dark followed by washes with PBS. NANOG protein was detected in cells fixed with 4% paraformaldehyde/PBS (15 min at room temperature) and washed with PBS. The cells were permeabilized using ice-cold methanol (−20°C for 10 min), washed with PBS, and incubated with blocking solution (PBS/0.3% Triton X-100/10% goat serum) for 1 h at room temperature. NANOG primary antibody (Abcam, 80892, 1:100) was diluted in blocking solution and applied overnight at 4°C followed by washes with PBST (PBS + 0.3% TritonX100). Secondary antibody (goat anti-rabbit IgG, 1:1000) was diluted in blocking solution and applied for 1 h at room temperature in the dark. The cells were stained with DAPI as described above.

Flow Cytometry and FACS

The parameters for gating cell populations by flow cytometry were established using the EGFP-negative parental DAK31 cells. EGFP fluorescence was measured in single-cell suspensions of ESCs in N2B27 medium on a BD FACSCalibur or BD LSR Fortessa. Cell populations or single cells were sorted in 2iL using a BD FACSAria III cell sorter. Single cells were sorted into wells of a 96-well plate coated with mouse fibroblasts and containing 2iL medium.

qRT-PCR

RNA (1 μg), purified using an RNeasy Mini Kit (QIAGEN) was used to synthesize cDNA using SuperScript First-Strand Synthesis System (Invitrogen). Approximately 1/60 of the cDNA was amplified using a Platinum SYBR Green QPCR kit (Invitrogen) under the

following conditions: 50°C for 2 min, then 95°C for 2 min followed by 40 cycles of 95°C for 15 s, then 60°C for 30 s, with a final cycle consisting of 95°C for 1 min, 60°C for 30 s, and 95°C for 15 s. Primer sequences are in Table S4.

Single-Cell cDNA Library Preparation and Sequencing

Rex1-EGFP cells maintained in 2iL were sorted into single cells using a BD FACSJazz cell sorter. Wild-type DAK31 parental cells (Blair et al., 2012) were used as a control to exclude EGFP-negative and auto-fluorescent cells. CF-1 fibroblast cells were used to exclude feeder cell contamination. Finally, E3 *Rex1*-EGFP cells were sorted into three populations, $EGFP^{high}$, $EGFP^{low}$, and $EGFP^{neg}$, and single cells plated into a 96-well plate containing 2 μL of lysis buffer. To allow for batch effects, triplicate plates were generated in which 32 cells from each group were plated per plate. A population of 2×10^4 $EGFP^{low}$ cells were also plated into a 2 cm^2 well and maintained on CF-1 feeders, in 2iL for 4 days prior to flow cytometry analysis. cDNA from single cells was generated using the smart-seq2 protocol (Picelli et al., 2014). Illumina Nextera reagents were used for library construction and the library was sequenced on the HiSeq 4000 Sequencing System at 75PE to yield a minimum of 290M+290M reads. The single-cell sequencing data are available at Edinburgh Datashare (https://doi.org/10.7488/ds/2639).

Statistical Analysis

Unless specified, all experiments were performed on triplicate biological or experimental samples. The data presented represented means ± SD or SEM. Single-cell sequencing data were analyzed using R to generate principal-component analysis (PCA) and box plots.

SUPPLEMENTAL INFORMATION

Supplemental Information can be found online at https://doi.org/10.1016/j.stemcr.2019.12.001.

AUTHOR CONTRIBUTIONS

S.M., J.W., A.J., and T.B. conceived and designed the study. S.M., J.W., T.O., J.O., L.S., and T.W. performed the study. T.C. and A.S. planned and performed the single-cell RNA sequencing experiments. D.F.C. constructed the β-catenin TALENs. S.M., J.W., A.J., T.C., and T.B. analyzed the data. S.M. and T.B. wrote the manuscript. All authors reviewed the manuscript.

ACKNOWLEDGMENTS

The authors would like to express their sincere gratitude to Mr William Mungall, Mrs Julie Thompson, and Mrs Ailsa Travers for their excellent technical support at the Biomedical Research Resources at the University of Edinburgh; Professor Austin Smith and Dr Kathryn Blair, University of Cambridge, for providing the DAK31 cell line; Dr Tetsushi Sakuma and Professor Takashi Yamamoto, Hiroshima University, for guidance in TALEN construction; and Mr Colin Simpson for curation of data files. This work was supported by funding from the Biotechnology and Biological Sciences Research Council Institute Strategic Programme grants BB/J004316/1, BB/J004332/1; BBSRC Response mode grant BB/H012478/1, BB/M023397/1; and European Community's Seventh Framework Program (FP7/2007-2013) under grant agreement no. HEALTH-F4-2010-241504 (EURATRANS).

Received: August 15, 2019
Revised: November 29, 2019
Accepted: December 2, 2019
Published: January 2, 2020

REFERENCES

Bao, L., He, L., Chen, J., Wu, Z., Liao, J., Rao, L., Ren, J., Li, H., Zhu, H., Qian, L., et al. (2011). Reprogramming of ovine adult fibroblasts to pluripotency via drug-inducible expression of defined factors. Cell Res. *21*, 600–608.

Bhatia, S., Pilquil, C., Roth-Albin, I., and Draper, J.S. (2013). Demarcation of stable subpopulations within the pluripotent hESC compartment. PLoS One *8*, e57276.

Blair, K., Leitch, H.G., Mansfield, W., Dumeau, C.-E., Humphreys, P., and Smith, A.G. (2012). Culture parameters for stable expansion, genetic modification and germline transmission of rat pluripotent stem cells. Biol. Open *1*, 58–65.

Boroviak, T., Loos, R., Bertone, P., Smith, A., and Nichols, J. (2014). The ability of inner-cell-mass cells to self-renew as embryonic stem cells is acquired following epiblast specification. Nat. Cell Biol. *16*, 516–528.

Buehr, M., Nichols, J., Stenhouse, F., Mountford, P., Greenhalgh, C.J., Kantachuvesiri, S., Brooker, G., Mullins, J., and Smith, A.G. (2003). Rapid loss of Oct-4 and pluripotency in cultured rodent blastocysts and derivative cell lines. Biol. Reprod. *68*, 222–229.

Buehr, M., Meek, S., Blair, K., Yang, J., Ure, J., Silva, J., McLay, R., Hall, J., Ying, Q.L., and Smith, A. (2008). Capture of authentic embryonic stem cells from rat blastocysts. Cell *135*, 1287–1298.

Chen, Y., Blair, K., and Smith, A. (2013). Robust self-renewal of rat embryonic stem cells requires fine-tuning of glycogen synthase kinase-3 inhibition. Stem Cell Rep. *1*, 209–217.

Doetschman, T., Gregg, R.G., Maeda, N., Hooper, M.L., Melton, D.W., Thompson, S., and Smithies, O. (1987). Targetted correction of a mutant HPRT gene in mouse embryonic stem cells. Nature *330*, 576–578.

Evans, M.J., and Kaufman, M. (1981). Establishment in culture of pluripotential cells from mouse embryos. Nature *292*, 154–156.

Gao, X., Nowak-Imialek, M., Chen, X., Chen, D., Herrmann, D., Ruan, D., Chen, A.C.H., Eckersley-Maslin, M.A., Ahmad, S., Lee, Y.L., et al. (2019). Establishment of porcine and human expanded potential stem cells. Nat. Cell Biol. *21*, 687–699.

Garcia-Tuñon, I., Guallar, D., Alonso-Martin, S., Benito, A.A., Benítez-Lázaro, A., Pérez-Palacios, R., Muniesa, P., Climent, M., Sánchez, M., Vidal, M., et al. (2011). Association of Rex-1 to target genes supports its interaction with Polycomb function. Stem Cell Res. *7*, 1–16.

Guallar, D., Pérez-Palacios, R., Climent, M., Martínez-Abadía, I., Larraga, A., Fernández-Juan, M., Vallejo, C., Muniesa, P., and Schoorlemmer, J. (2012). Expression of endogenous retroviruses is negatively regulated by the pluripotency marker Rex1/Zfp42. Nucleic Acids Res. *40*, 8993–9007.

Hooper, M., Hardy, K., Handyside, A., Hunter, S., and Monk, M. (1987). HPRT-deficient (Lesch-Nyhan) mouse embryos derived from germline colonization by cultured cells. Nature *326*, 292–295.

Hosler, B.A., LaRosa, G.J., Grippo, J.F., and Gudas, L.J. (1989). Expression of REX-1, a gene containing zinc finger motifs, is rapidly reduced by retinoic acid in F9 teratocarcinoma cells. Mol. Cell. Biol. *9*, 5623–5629.

Kalkan, T., Olova, N., Roode, M., Mulas, C., Lee, H.J., Nett, I., Marks, H., Walker, R., Stunnenberg, H.G., Lilley, K.S., et al. (2017). Tracking the embryonic stem cell transition from ground state pluripotency. Development *144*, 1221–1234.

Kim, J.D., Faulk, C., and Kim, J. (2007). Retroposition and evolution of the DNA-binding motifs of YY1, YY2 and REX1. Nucleic Acids Res. *35*, 3442–3452.

Kim, J.D., Kim, H., Ekram, M.B., Yu, S., Faulk, C., and Kim, J. (2011). Rex1/Zfp42 as an epigenetic regulator for genomic imprinting. Hum. Mol. Genet. *20*, 1353–1362.

Kirschner, K., Chandra, T., Kiselev, V., Flores-Santa Cruz, D., Macaulay, I.C., Park, H.J., Li, J., Kent, D.G., Kumar, R., Pask, D.C., et al. (2017). Proliferation drives aging-related functional decline in a subpopulation of the hematopoietic stem cell compartment. Cell Rep. *19*, 1503–1511.

Li, P., Tong, C., Mehrian-Shai, R., Jia, L., Wu, N., Yan, Y., Maxson, R.E., Schulze, E.N., Song, H., Hsieh, C.-L., et al. (2008). Germline competent embryonic stem cells derived from rat blastocysts. Cell *135*, 1299–1310.

Martin, G.R. (1981). Isolation of a pluripotent cell line from early mouse embryos cultured in medium conditioned by teratocarcinoma stem cells. Proc. Natl. Acad. Sci. U S A *78*, 7634–7638.

Masui, S., Ohtsuka, S., Yagi, R., Takahashi, K., Ko, M.S.H., and Niwa, H. (2008). Rex1/Zfp42 is dispensable for pluripotency in mouse ES cells. BMC Dev. Biol. *8*, 45.

Meek, S., Buehr, M., Sutherland, L., Thomson, A., Mullins, J.J., Smith, A.J., and Burdon, T. (2010). Efficient gene targeting by homologous recombination in rat embryonic stem cells. PLoS One *5*, e14225.

Meek, S., Wei, J., Sutherland, L., Nilges, B., Buehr, M., Tomlinson, S.R., Thomson, A.J., and Burdon, T. (2013). Tuning of beta-catenin activity is required to stabilize self-renewal of rat embryonic stem cells. Stem Cells *31*, 2104–2115.

Meek, S., Mashimo, T., and Burdon, T. (2017). From engineering to editing the rat genome. Mamm. Genome *28*, 302–314.

Miyanari, Y., and Torres-Padilla, M.-E. (2012). Control of ground-state pluripotency by allelic regulation of Nanog. Nature *483*, 470–473.

Navarro, P., Oldfield, A., Legoupi, J., Festuccia, N., Dubois, A., Attia, M., Schoorlemmer, J., Rougeulle, C., Chambers, I., and Avner, P. (2010). Molecular coupling of Tsix regulation and pluripotency. Nature *468*, 457–460.

Nichols, J., and Smith, A. (2009). Naive and primed pluripotent states. Cell Stem Cell *4*, 487–492.

Pelton, T.A., Sharma, S., Schulz, T.C., Rathjen, J., and Rathjen, P.D. (2002). Transient pluripotent cell populations during primitive ectoderm formation: correlation of in vivo and in vitro pluripotent cell development. J. Cell Sci. *115*, 329–339.

Picelli, S., Faridani, O.R., Björklund, A.K., Winberg, G., Sagasser, S., and Sandberg, R. (2014). Full-length RNA-seq from single cells using Smart-seq2. Nat. Protoc. *9*, 171–181.

Rezende, N.C., Lee, M.-Y., Monette, S., Mark, W., Lu, A., and Gudas, L.J. (2011). Rex1 (Zfp42) null mice show impaired testicular function, abnormal testis morphology, and aberrant gene expression. Dev. Biol. *356*, 370–382.

Rodríguez, A., Allegrucci, C., and Alberio, R. (2012). Modulation of pluripotency in the porcine embryo and iPS cells. PLoS One *7*, e49079.

Rogers, M.B., Hosler, B.A., and Gudas, L.J. (1991). Specific expression of a retinoic acid-regulated zinc finger gene, Rex-1, in preimplantation embryos, trophoblast and spermatocytes. Development *113*, 815–824.

Thomas, K.R., and Capecchi, M.R. (1987). Site-directed mutagenesis by gene targeting in mouse embryo-derived stem cells. Cell *51*, 503–512.

Tong, C., Li, P., Wu, N.L., Yan, Y., and Ying, Q.L. (2010). Production of p53 gene knockout rats by homologous recombination in embryonic stem cells. Nature *467*, 211–213.

Toyooka, Y., Shimosato, D., Murakami, K., Takahashi, K., and Niwa, H. (2008). Identification and characterization of subpopulations in undifferentiated ES cell culture. Development *135*, 909–918.

Wray, J., Kalkan, T., Gomez-Lopez, S., Eckardt, D., Cook, A., Kemler, R., and Smith, A. (2011). Inhibition of glycogen synthase kinase-3 alleviates Tcf3 repression of the pluripotency network and increases embryonic stem cell resistance to differentiation. Nat. Cell Biol. *13*, 838–845.

Ying, Q.L., Wray, J., Nichols, J., Batlle-Morera, L., Doble, B., Woodgett, J., Cohen, P., and Smith, A. (2008). The ground state of embryonic stem cell self-renewal. Nature *453*, 519–523.

Stem Cell Reports, Volume *14*

Supplemental Information

A Stem Cell Reporter for Investigating Pluripotency and Self-Renewal in the Rat

Stephen Meek, Jun Wei, Taeho Oh, Tom Watson, Jaime Olavarrieta, Linda Sutherland, Daniel F. Carlson, Angela Salzano, Tamir Chandra, Anagha Joshi, and Tom Burdon

Figure S1. (Related to Figure 1)

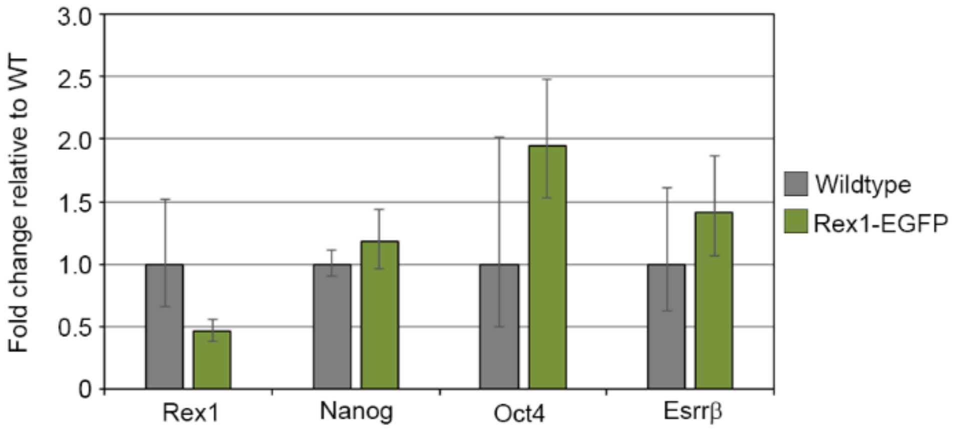

Figure S1. ESC gene expression in heterozygous *Rex1*-EGFP ESCs. qRT-PCR analysis for *Rex1* and the core ESC transcription factors Nanog, Oct4 and Esrrb in wild type (WT) and heterozygous *Rex1*-EGFP rESCs (mean and SD of three WT and four HET biological replicates).

Figure S2. (Related to Figure 2)

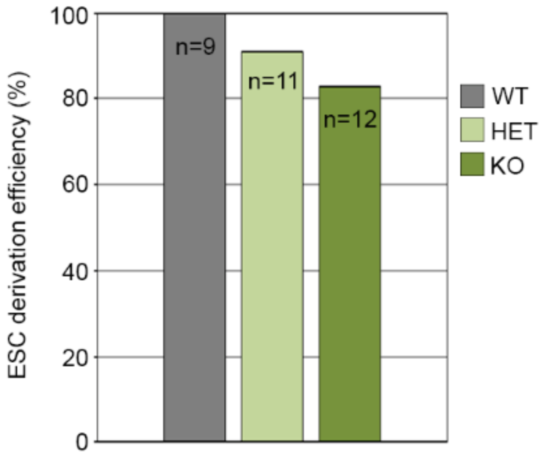

Figure S2. Derivation efficiency of *Rex1*-EGFP ESC lines.
Derivation efficiency of wild type (WT), heterozygous (HET) and knockout (KO) *Rex1*-EGFP ESCs from a total of nine, eleven and twelve E4.5 blastocysts respectively (all genotypes were derived from a total of four crosses).

Figure S3. (Related to Figure 4)

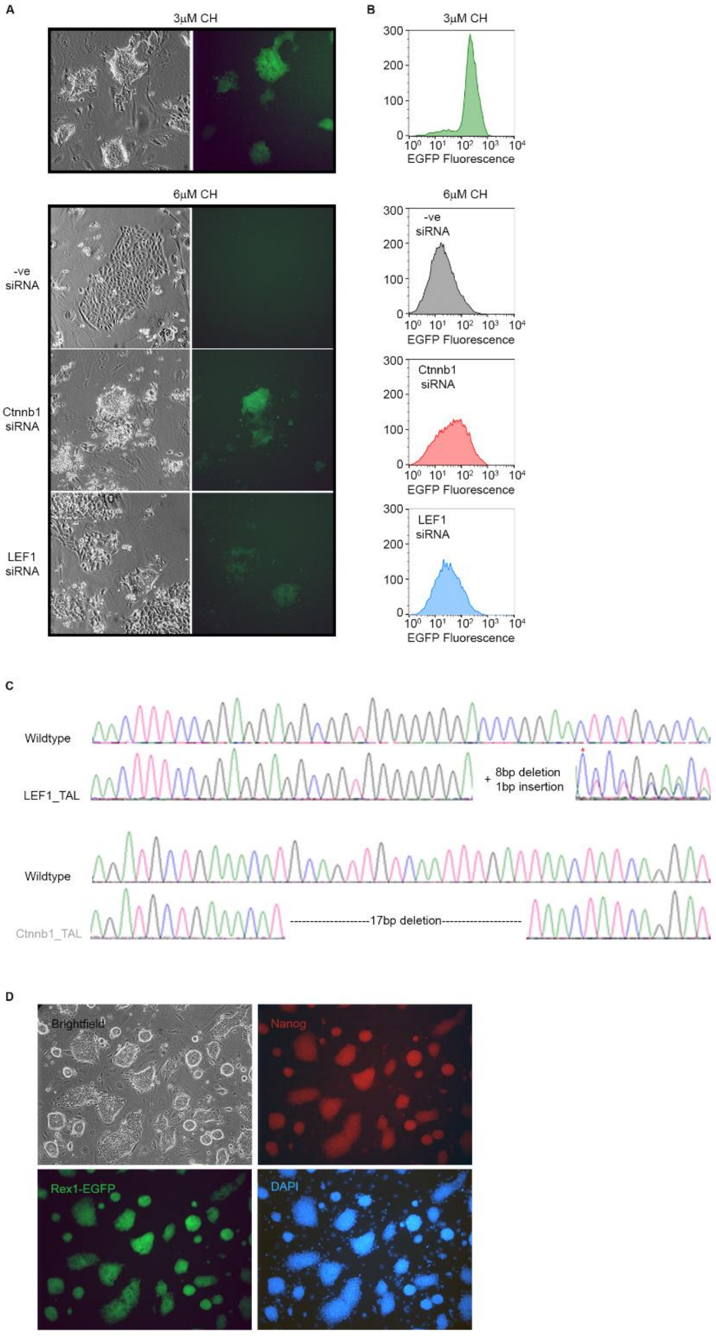

Figure S3. Sensitivity of *Rex1*-EGFP reporter during CHIR-induced differentiation and siRNA-mediated rescue. (A) Brightfield and fluorescent images of *Rex1*-EGFP rat ESCs cultured for four days in normal (3mM) and differentiating (6mM) rESC culture conditions following transfection with control (-ve), Ctnnb1 or LEF1 siRNAs. (magnification x100). (B) Flow cytometry analysis of *Rex1*-EGFP rESCs cultured for four days in normal (3mM) and differentiating (6mM) rESC culture conditions following transfection with control (-ve), Ctnnb1 or LEF1 siRNAs. (C) Sequence chromatograms of genomic DNA from wild type and TALEN-modified Ctnnb1 and LEF1 rESC clones showing frameshift-modified alleles (red asterisk highlights inserted base). (D) Brightfield, fluorescent (EGFP) and immunostained (Nanog) images of *Rex1*-EGFP rESC colonies cultured in 2iL. DAPI-stained cells are also shown. (magnification x100).

Figure S4. (Related Figure 5)

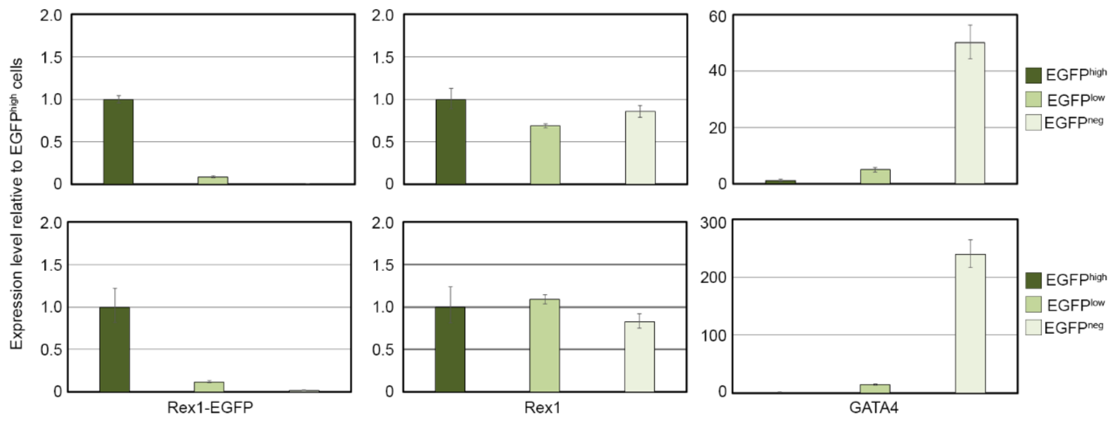

Figure S4. qRT-PCR analysis of FAC-sorted *Rex1*-EGFP rESCs.
qRT-PCR analysis for *Rex1*-EGFP, *Rex1* and *Gata4* in FAC-sorted *Rex1*-EGFP high, low and negative cells from two independent rESC lines (mean and SD of three technical replicates).

Figure S5. (Related to Figure 6)

A

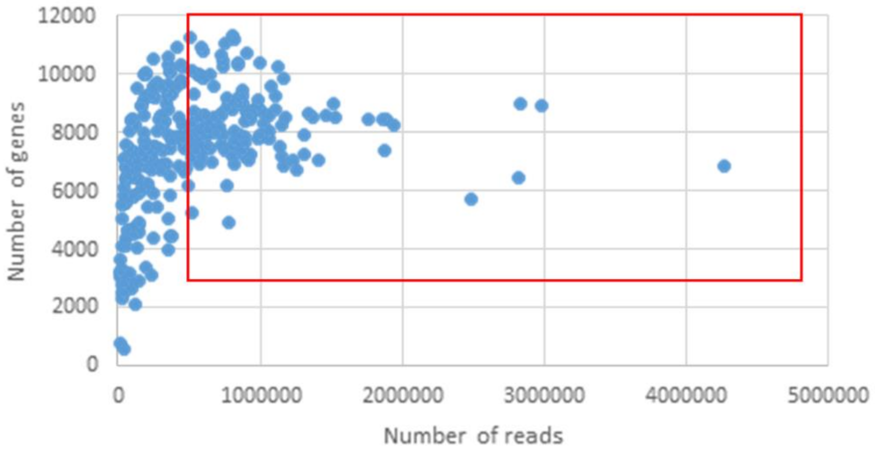

B

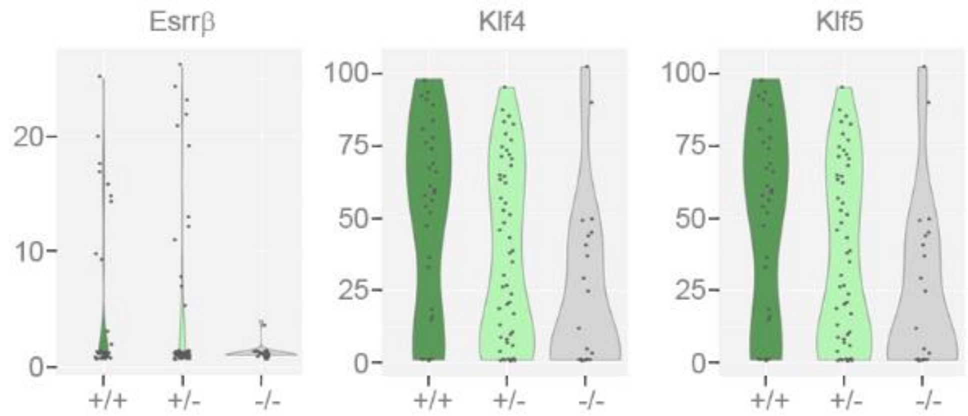

Figure S5. Single cell expression analysis of rESC. (A) Single cell RNA sequence quality control selection. Plot depicting the number of reads against the number of genes for each single cell sample. The cells in the red box were selected for downstream analysis. (B) Violin plots of *Esrrb*, *Klf4* and *Klf5* expression in *Rex1*$^{mRNA+/EGFP+,}$ *Rex1*$^{mRNA+/EGFP-}$ and *Rex1*$^{mRNA-/EGFP-}$ cells.

Figure S6. (Related to Figure 6)

A

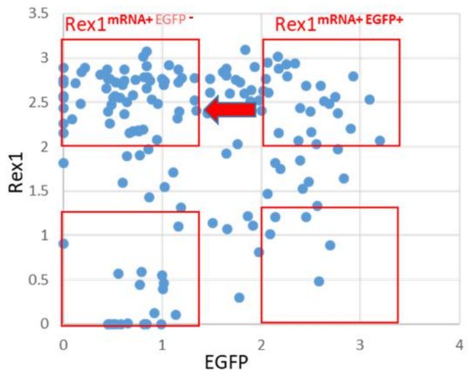

B

DOWN-REGULATED: Rps12, Fam120b, Zbtb45, Ube2s, Tmem160, Zfp428, Egln2, Hnrnpl, Spint2, Emc10, Ptov1, Nucb1, Prmt3, LOC100912195, Igf1r, Bnc1, Nup98, Taf10, Eif3f, Ubfd1, Plk1, Xpo6, Mapk3, Mvp, Utf1, Deaf1, Dhcr7, Dhcr7, Tmem134, Fam89b, Scyl1, Stx5, Tmem179b, Ints5, Incenp, Carnmt1, Uhrf2, Fam169a, LOC103689968, Hdgf, Hist2h2aa2, Hist2h2aa3, Ddx20, Gclm, Ssx2ip, Zzz3, Armc10, Bpgm, Slc25a26, Ptms, Rpl8, Qsox2, Ttf1, LOC100912604, Celf1, Atg13, Dnajc17, Ndufaf5, Map1lc3a, Scand1, Dnttip1, Dnttip1, Ube2c, Prpf6, Nudt2, Arhgef39, Clta, Igfbpl1, LOC108351137, Tmem39b, Cep85, Pink1, Ctnnbip1, Prkcz, Maoa, Dkc1, Fez2, Gckr, Ppm1g, Rps10l1, Baz1a, Rpl6-ps1, Glrx5, Crip1, Myl6, Fzr1, Gadd45b, Sppl2b, Sf3a2, Med16, Zfp472, Ccdc59, Gns, Shmt2, Trmt12, Grina, Gpaa1, Fbxl6, Pim3, Zcrb1, LOC688906, Tuba1c, Atg101, Atg4d, Ilf3, Rp9, Sidt2, Alas1, Smarcc1, Rpsa, Rpl36, Safb, Mpnd, Tjap1, Tmem63b, Ptpn18, Rnf149, Tex30, Sgo2, RGD1562399, Mreg, Cnppd1, Abcb6, Wdfy1, Ilkap, Crlf2, Tmem165, LOC257642, LOC257642, Ctbp1, Rasl10a, Mrps24, Nudcd3, Cobl, Wdr92, Insig2, Fmod, Uhmk1, Psen2, Tmem63a, Pdpk1, Eme2, Gnptg, Chtf18, Fam173a, Jmjd8, Stub1, Hba-a3, Hba-a2, Mpg, Ergic1, RGD1564698, Hnrnpab, Cdkn2aipnl, Rnf187, LOC108348082, Sco1, Aurkb, C1qbp, Ube2g1, Spag5, Trim25, Fam117a, Top2a, Nt5c3b, Rab5c, Lsm12, Cdc42ep4, Recql5, Galk1, Slc38a10, Faap100, Mafg, LOC108348081, Ccnb1ip1, Zfp219, Lrp10, Psmb5, Cdca2, Wdfy2, R3hcc1, Polr3d, B4galt7, Rab24, Tspan17, AC120310.1, LOC679342, Mcur1, Lyrm4, Hist1h1d, Hist1h3b, Hist1h1b, Wac, Rbm17, Ccser2, Ocel1, Fkbp8, Sugp2, Armc6, Gapdh-ps2, Dusp26, Coprs, Upf3a, Lamp1, Osbpl11, Cep19, Slc25a1, Rock1, Sil1, Ccdc112, Heatr3, Ccdc130, RGD1564093, Prmt9, Ctcf, Pskh1, Prmt7, Cog8, Aars, Gtf2h4, RT1-CE16, Ddah2, Bag6, Kifc1, RGD1564450, Snrpc, Trappc5, Gtf3a, Rac1, Cops6, Tmem248, Ccdc92, Plbd2, Gcn1l1, Unc119b, Iscu, Pitpnb, Chchd10, Tbx3, eGFP. **UP-REGULATED**: Ndufa4, Fubp3, LOC102557117, Prdx1, Srsf7, Map3k12, Sumo1, H3f3b, Calm2, Jund, Naa50, LOC102550729, Bud31, Mt-nd2

C

GO: Biological Process

	ID	Name	Source	pValue	Bonferroni	Genes from Input	Genes in Annotation
1	GO:0051276	chromosome organization		1.354E-8	4.491E-5	39	1189
2	GO:0007059	chromosome segregation		7.488E-7	2.483E-3	17	333
3	GO:0016071	mRNA metabolic process		6.975E-6	2.313E-2	23	664

D

Chromosome organisation GO:0051276

	Entrez Gene ID	Gene Symbol	Gene Name
1	3619	INCENP	inner centromere protein
2	55352	COPRS	coordinator of PRMT5 and differentiation stimulator
3	7270	TTF1	transcription termination factor 1
4	7786	MAP3K12	mitogen-activated protein kinase kinase kinase 12
5	51322	WAC	WW domain containing adaptor with coiled-coil
6	646	BNC1	basonuclin 1
7	8337	HIST2H2AA3	histone cluster 2 H2A family member a3
8	6294	SAFB	scaffold attachment factor B
9	8358	HIST1H3B	histone cluster 1 H3 family member b
10	9400	RECQL5	RecQ like helicase 5
11	1736	DKC1	dyskerin pseudouridine synthase 1
12	151246	SGO2	shugoshin 2
13	54496	PRMT7	protein arginine methyltransferase 7
14	6881	TAF10	TATA-box binding protein associated factor 10
15	5347	PLK1	polo like kinase 1
16	115426	UHRF2	ubiquitin like with PHD and ring finger domains 2
17	10980	COPS6	COP9 signalosome subunit 6
18	7917	BAG6	BCL2 associated athanogene 6
19	3833	KIFC1	kinesin family member C1
20	11065	UBE2C	ubiquitin conjugating enzyme E2 C
21	4928	NUP98	nucleoporin 98
22	723790	HIST2H2AA4	histone cluster 2 H2A family member a4
23	80218	NAA50	N(alpha)-acetyltransferase 50, NatE catalytic subunit
24	10615	SPAG5	sperm associated antigen 5
25	415116	PIM3	Pim-3 proto-oncogene, serine/threonine kinase
26	2968	GTF2H4	general transcription factor IIH subunit 4
27	10664	CTCF	CCCTC-binding factor
28	11177	BAZ1A	bromodomain adjacent to zinc finger domain 1A
29	3007	HIST1H1D	histone cluster 1 H1 family member d
30	3009	HIST1H1B	histone cluster 1 H1 family member b
31	6599	SMARCC1	SWI/SNF related, matrix associated, actin dependent regulator of chromatin subfamily c member 1
32	3021	H3F3B	H3 histone family member 3B
33	1487	CTBP1	C-terminal binding protein 1
34	10196	PRMT3	protein arginine methyltransferase 3
35	5595	MAPK3	mitogen-activated protein kinase 3
36	57820	CCNB1IP1	cyclin B1 interacting protein 1
37	7153	TOP2A	DNA topoisomerase II alpha
38	65018	PINK1	PTEN induced kinase 1
39	9212	AURKB	aurora kinase B

E

Chromosome segregation GO:0007059

	Entrez Gene ID	Gene Symbol	Gene Name
1	3619	INCENP	inner centromere protein
2	157313	CDCA2	cell division cycle associated 2
3	9400	RECQL5	RecQ like helicase 5
4	151246	SGO2	shugoshin 2
5	5347	PLK1	polo like kinase 1
6	7917	BAG6	BCL2 associated athanogene 6
7	3833	KIFC1	kinesin family member C1
8	64793	CEP85	centrosomal protein 85
9	11065	UBE2C	ubiquitin conjugating enzyme E2 C
10	4928	NUP98	nucleoporin 98
11	80218	NAA50	N(alpha)-acetyltransferase 50, NatE catalytic subunit
12	10615	SPAG5	sperm associated antigen 5
13	10664	CTCF	CCCTC-binding factor
14	63922	CHTF18	chromosome transmission fidelity factor 18
15	57820	CCNB1IP1	cyclin B1 interacting protein 1
16	7153	TOP2A	DNA topoisomerase II alpha
17	9212	AURKB	aurora kinase B

Figure S6. Genes differentially expressed between *Rex1*$^{\mathrm{mRNA+EGFP+}}$ and *Rex1*$^{\mathrm{mRNA+EGFP-}}$ cell populations. (**A**) Scatter plot identifying the *Rex1*$^{\mathrm{mRNA+EGFP+}}$ and *Rex1*$^{\mathrm{mRNA+EGFP-}}$ cell populations**.** (**B**) List of genes differentially expressed between the *Rex1*$^{\mathrm{mRNA+EGFP+}}$ and *Rex1*$^{\mathrm{mRNA+EGFP-}}$ cells. (**C**) Three biological processes most highly enriched in the differentially expressed genes. (**D**) List of differentially expressed genes associated with chromosome organisation. (**E**) List of differentially expressed genes associated with chromosome segregation.

Table S1. Chimaera formation and germline transmission efficiency. (Related to Figure 1)

Cell line	Genotype	Pups born	Chimaeras	GLT transmission
DAK31	Wild type	62	10M/11F (34%)	4/10 (40%)
E3	targeted	32	7M/6F (41%)	2/7 (29%)

Table S2. Viability of *Rex1* null rats. (Related to Figure 1)

Pups born	Wild type	Heterozyous	Homozygous
205[#]	41 (20%)	122 (60%)	42 (20%)

[#] pups born from six breeding pairs.

Table S3. *Rex1* null male rat fertility. (Related to Figure 1)

Male genotype	Stage	No. of litters	No. of embryos	Av. Litter size
Wild type (n=3)	Mid-gestation	5	40	8.0
Wild type (n=2)	E4.5	3	10	3.3
	Total	8	50	6.2
Rex1-null (n=4)	Mid-gestation	5	28	5.6
Rex1-null (n=4)	E4.5	4	27	6.7
	Total	9	55	6.1

Table S4. qRT-PCR primer list. (Related to Experimental Procedures)

**EGFP Forward**	**ACGTAAACGGCCACAAGTTC**
**EGFP Reverse**	**AAGTCGTGCTGCTTCATGTG**
**Esrrb Forward**	**AGGCTTGCAAGGCGTTCTT**
**Esrrb Reverse**	**AGGACTTGCGCCTCCGTTT**
**Gapdh Forward**	**ATGACTCTACCCACGGCAAG**
**Gapdh Reverse**	**TGGGTTTCCCGTTGATGACC**
**Gata4 Forward**	**GCGGCCTCTACATGAAGCTC**
**Gata4 Forward**	**TTCCGTTTTCTGGTTTGAATC**
**Nanog Forward**	**TACCTCAGCCTCCAGCAGAT**
**Nanog Reverse**	**GCAATGGATGCTGGGATACT**
**Oct4 Forward**	**GAAGTTGGAGAAGGTGGAACC**
**Oct4 Reverse**	**GTGTACCCCAAGGTGATCCTC**
**Rex1 Forward**	**GATTTCAACTTGCGCACCC**
**Rex1 Reverse**	**CTCTTCTCGCAGCCATCAAAA**
**Sox2 Forward**	**AACCCCAAGATGCACAACTC**
**Sox2 Reverse**	**CTTGGCCTCGTCGATGAAC**
**Tsix Forward**	**GTATCCACAGCCCCGATG**
**Tsix Reverse**	**ACCTCGGATACCTGCGTTT**

Table S5. Number of *Rex1*-EGFP FAC-sorted single cells selected for each population after quality control. (Related to Figure 6)

Plate	$EGFP^{neg}$	$EGFP^{low}$	$EGFP^{high}$
1	16(32)	28(32)	8(32)
2	23(32)	27(32)	10(32)
3	10(32)	16(32)	13(32)
4#	43(96)		

() Total number of cells collected.

Replated *Rex1*-$EGFP^{low}$ cells.

Table S6. Number of cells selected based on EGFP fluorescence and mRNA expression (*Rex1* and EGFP). (Related to Figure 6)

	$EGFP^{neg}$	$EGFP^{low}$	$EGFP^{high}$	$EGFP^{low\text{-}replated}$
Rex1$^{mRNA+/EGFP+}$ (35)	2	19	14	0
Rex1$^{mRNA+/EGFP-}$ (78)	15	37	5	22
Rex1$^{mRNA-/EGFP-}$ (24)	11	0	1	12

Supplemental Experimental Procedures

Chimaera Generation

Rat blastocysts at E4.5 days post-coitum were collected by noon on the day of injection and cultured for 2–3 hours in KSOM embryo culture medium to ensure cavitation. Cells were disaggregated in TVP, pelleted in N2B27 and pre-plated on gelatin-coated tissue culture plastic in 2iL for 45-60 minutes. Non-attached cells were pelleted and resuspended in N2B27 containing 20mM HEPES buffer then kept on ice prior to injection. Blastocysts were injected with 10–12 cells, then transferred into the uteri of pseudopregnant Sprague Dawley rats.

Genotyping *Rex1*-EGFP rats

150ng of genomic DNA was amplified using a single PCR reaction containing three oligonucleotides designed to identify the wild type (REX5'interFOR2–GTGGATGTCAGGACAATCTGAG, REX5'wtREV2-CCACTTGTCTTTGCCATTTTCT) and targeted (REX5'interFOR2-GTGGATGTCAGGACAATCTGAG, REXgfpREV–GGACTTGAAGAAGTCGTGCTG) *Rex1* alleles. The PCR was performed using NEB Q5 HotStart Taq Polymerase under the following conditions; 98°C for 1 minute, followed by 32 cycles of 98°C for 10s, 65°C for 30s and 72°C for 1 minute with a final extension of 72°C for 10 minutes. Products were visualised with ethidium bromide on a 2% TAE agarose gel. Expected sizes for wild-type and targeted alleles are 292bp and 524bp respectively.

Sexing rats and rESCs

150ng of genomic DNA was amplified using a single PCR reaction containing four oligonucleotides. The presence of a Y chromosome was determined using oligonucleotides designed to the rat *Sry* gene (For-AAGCCTTACAGAAGCCGAAA, Rev-TACAGTTTTGTTGAGGCAACT) and oligonucleotides designed to the rat *Sox2* gene (For-ATGATGGAGACGGAGCTGAA, Rev-CTCCGGGAAGCGTGTACTTA) were used as an internal control. The PCR was performed using NEB Q5 HotStart Taq Polymerase under the following conditions; 98°C for 1 minute, followed by 32 cycles of 98°C for 10s, 65°C for 30s and 72°C for 30s with a final extension of 72°C for 10 minutes. Products were visualised with ethidium bromide on a 2% TAE agarose gel. Expected sizes for *Sry* and *Sox2* PCR products are 165bp and 385bp respectively.

Southern blotting

Eight to ten micrograms of genomic DNA were digested with 200 units of restriction enzyme at 37°C for 30 hours. The resulting DNA fragments were resolved on a 0.7% TAE agarose gel overnight at 25V. The DNA fragments were UV-nicked prior to transfer to Hybond N + Nylon membrane (GE Healthcare, RPN203B) as described in the manufacturer's instructions. Following transfer, the DNA was UV cross-linked on to the membrane. Probes were prepared by PCR amplification of *Rex1* sequence flanking the homology arms to generate a 595pb 5' probe and a 360bp 3' probe (5' probe sequence TAGAATTCATAAGACGTTGG to TTTGGCATGCACTTATTTA and 3' probe sequence TTCCACCCCCTCCCTCATGC to TGGTTACATTGTGTTTCTGC). 25ng of probe DNA was radioactively labelled with α–dCTP P^{32} using High Prime (Roche, 11 585 592 001), then hybridised to the membrane overnight at 65°C in Church solution containing 10μg/ml sonicated Herring Sperm DNA and 10μg/ml tRNA. Non-specifically bound probe was removed by washing in 2xSSC/0.1% (w/v) SDS at 65°C. The membrane was exposed to Kodak Biomax MS film at -80°C.

TALEN construction

The *β-catenin* TALEN pair (left target – GCTGAAACATGCAGTT, right target - ATCAGGATGACGCGG) were designed to exon 3 and kindly generated and supplied by Dan Carlson (Recombinetics, Inc.). The *Lef1* TALEN pair (left target – CCGGAGGAGGCGGTG, right target - TGCGCCACCGATGAG) were designed to exon 1 and generated by Golden Gate cloning. All TALENs were designed using the TALE-NT software (https://tale-nt.cac.cornell.edu/node/add/talen) and assembled using methods described in Sakuma et al. The TALENs were cloned into pCAG-T7 TALEN (Sangamo)-FokI-ELD-Destination and pCAG-T7-TALEN (Sangamo)-FokI-KKR-Destination expression plasmids. Rat ESCs were transfected at a density of 0.5x10^{4}/cm^{2} with 240ng left and right TALEN expression plasmid using Lipofectamine LTX (2.25μl LTX + 0.75μl PLUS reagent, Invitrogen) according to manufacturer's instructions. Transfection reagents were removed 16 hours post-transfection and the cells cultured for a further 3 days prior to imaging and flow cytometry analysis.

siRNA knockdown

Rat ESCs were transfected at a density of 0.5x10^{4}/cm^{2} with *Ctnnb1* (Ambion, s136459), *Lef1* (s139217) or negative control (Ambion, 4390846) siRNA at a final concentration of 50nM using Lipofectamine LTX (2.25μl

LTX + 0.75μl PLUS reagent, Invitrogen) according to manufacturer's instructions. Transfection reagents were removed 16 hours post-transfection and the cells cultured for a further 3 days prior to imaging and flow cytometry analysis.

Bioinformatic Analysis

The sequence files were first checked through FASTQC and then the sequencing reads were mapped to rat genome assembly Rnor_6.0 using Kallisto aligner (Bray et al., 2016). The gene counts were generated using HT-seq. We used a stringent criterion to select individual cells based on the sequencing depth (over 500K reads) and the total number of genes (over 3000 genes) (Figure S6). This resulted into a total of 49 $EGFP^{neg}$, 71 $EGFP^{low}$ and 31 $EGFP^{high}$ cells selected for further downstream analysis (Table S5). We also validated that all selected cells were obtained using ERCC count and MT read filter as well. The data for selected samples was normalised using Scran (Lun et al., 2016). The batch correction was performed using ComBat. The differentially expressed gene sets were obtained using scde (Kharchenko et al., 2014). We noted the inconsistency between EGFP and *Rex1* mRNA expression across cells (Figure 6). We therefore grouped cells based on EGFP and *Rex1* mRNA expression into three groups $Rex1^{mRNA+/EGFP+}$ (log expression value > 2), $Rex1^{mRNA+/EGFP-}$ and $Rex1^{mRNA-/EGFP-}$(log expression value < 1.3). Table S6 shows the overlap of cells selected based on EGFP activity and mRNA expression (*Rex1* and EGFP) groupings. All analysis was performed in R including generation of PCA plots and box plots.

Gene list enrichment analysis was performed using the ToppFun application within the ToppGene Suite (https://toppgene.cchmc.org/).

Ethics statement

Animal work conformed to guidelines for animal husbandry according to the UK Home Office and approval by the Roslin Institute Animal Ethics Committee. Animals were naturally mated and sacrificed under schedule 1, procedures that do not require specific Home Office approval.

Supplemental References

Bray, N.L., Pimentel, H., Melsted, P., and Pachter, L. (2016). Near-optimal probabilistic RNA-seq quantification. Nat. Biotechnol. *34*, 525–527.

Kharchenko, P. V, Silberstein, L., and Scadden, D.T. (2014). Bayesian approach to single-cell differential expression analysis. Nat. Methods *11*, 740–742.

Lun, A.T.L., Bach, K., and Marioni, J.C. (2016). Pooling across cells to normalize single-cell RNA sequencing data with many zero counts. Genome Biol. *17*, 75.
